# A systematic review of fall prediction models for community-dwelling older adults: comparison between models based on research cohorts and models based on routinely collected data

**DOI:** 10.1093/ageing/afae131

**Published:** 2024-07-09

**Authors:** Noman Dormosh, Bob van de Loo, Martijn W Heymans, Martijn C Schut, Stephanie Medlock, Natasja M van Schoor, Nathalie van der Velde, Ameen Abu-Hanna

**Affiliations:** Department of Medical Informatics, Amsterdam UMC location University of Amsterdam, Amsterdam, The Netherlands; Amsterdam Public Health, Aging and Later Life & Methodology, Amsterdam, The Netherlands; Department of Epidemiology and Data Science, Amsterdam UMC location Vrije Universiteit Amsterdam, Amsterdam, The Netherlands; Amsterdam Public Health, Aging and Later Life, Amsterdam, The Netherlands; Department of Epidemiology and Data Science, Amsterdam UMC location Vrije Universiteit Amsterdam, Amsterdam, The Netherlands; Amsterdam Public Health, Methodology & Personalized Medicine, Amsterdam, The Netherlands; Department of Medical Informatics, Amsterdam UMC location University of Amsterdam, Amsterdam, The Netherlands; Department of Laboratory Medicine, Amsterdam UMC location Vrije Universiteit Amsterdam, Amsterdam, The Netherlands; Amsterdam Public Health, Methodology & Quality of Care, Amsterdam, The Netherlands; Department of Medical Informatics, Amsterdam UMC location University of Amsterdam, Amsterdam, The Netherlands; Amsterdam Public Health, Aging and Later Life & Methodology, Amsterdam, The Netherlands; Department of Epidemiology and Data Science, Amsterdam UMC location Vrije Universiteit Amsterdam, Amsterdam, The Netherlands; Amsterdam Public Health, Aging and Later Life, Amsterdam, The Netherlands; Amsterdam Public Health, Aging and Later Life, Amsterdam, The Netherlands; Department of Internal Medicine, Section of Geriatric Medicine, Amsterdam UMC location University of Amsterdam, Amsterdam, The Netherlands; Department of Medical Informatics, Amsterdam UMC location University of Amsterdam, Amsterdam, The Netherlands; Amsterdam Public Health, Aging and Later Life & Methodology, Amsterdam, The Netherlands

**Keywords:** accidental falls, prediction models, risk stratification tools, geriatric medicine, prospective cohorts, electronic health records, routinely collected data, systematic review, older people

## Abstract

**Background:**

Prediction models can identify fall-prone individuals. Prediction models can be based on either data from research cohorts (cohort-based) or routinely collected data (RCD-based). We review and compare cohort-based and RCD-based studies describing the development and/or validation of fall prediction models for community-dwelling older adults.

**Methods:**

Medline and Embase were searched via Ovid until January 2023. We included studies describing the development or validation of multivariable prediction models of falls in older adults (60+). Both risk of bias and reporting quality were assessed using the PROBAST and TRIPOD, respectively.

**Results:**

We included and reviewed 28 relevant studies, describing 30 prediction models (23 cohort-based and 7 RCD-based), and external validation of two existing models (one cohort-based and one RCD-based). The median sample sizes for cohort-based and RCD-based studies were 1365 [interquartile range (IQR) 426–2766] versus 90 441 (IQR 56 442–128 157), and the ranges of fall rates were 5.4% to 60.4% versus 1.6% to 13.1%, respectively. Discrimination performance was comparable between cohort-based and RCD-based models, with the respective area under the receiver operating characteristic curves ranging from 0.65 to 0.88 versus 0.71 to 0.81. The median number of predictors in cohort-based final models was 6 (IQR 5–11); for RCD-based models, it was 16 (IQR 11–26). All but one cohort-based model had high bias risks, primarily due to deficiencies in statistical analysis and outcome determination.

**Conclusions:**

Cohort-based models to predict falls in older adults in the community are plentiful. RCD-based models are yet in their infancy but provide comparable predictive performance with no additional data collection efforts. Future studies should focus on methodological and reporting quality.

## Key Points

- Fall prediction models using research cohort data (cohort-based) performed comparably to those using routinely collected data (RCD-based).
- We identified 23 cohort-based and 7 routinely collected data–based (RCD-based) prediction models for falls in community-dwelling older adults.
- All prediction models, except one cohort-based model, had high bias risk due to statistical analysis and fall determination.
- The vast majority of the included fall prediction models were poorly reported.
- Future studies should focus on improving the quality of methods and reporting.

## Introduction

Falls and fall-related injuries are common in older adults. One-third of adults aged ≥65 years falls at least once per year and one out of every five falls is injurious [1]. Even if non-injurious, falls can result in functional decline and avoidance of social and physical activities [2, 3]. Risk screening is an essential part of fall-preventive care pathways [4]. There are a growing number of studies that describe the development and validation of prediction models for falls, intended for identifying fall-prone patients and supporting clinical decisions. A prediction model is in essence a mathematical equation that relates multiple predictors for a particular individual to the probability of a (future) particular outcome [5]. Usually, the development of these prediction models is based on research cohorts although routinely collected health data (RCD) are increasingly being used [6]. RCD are collected for purposes other than research or without specific a priori research questions posed before collection [7]. Examples of RCD include electronic health records (EHRs) and health insurance administrative data. Although both are cohort studies, the term ‘cohort’ in this paper refers to research cohorts using data collected specifically for study purposes.

Todd *et al*. describe various theoretical advantages and disadvantages of using RCD as opposed to data from cohorts in research among older populations [8]. For example, routine data collection procedures minimise costs and burden on participants and may allow researchers to capture many clinical events in large samples across many time points [8]. Moreover, datasets derived from RCD can cover patient groups that would be otherwise difficult to enrol, such as the oldest-old or patients with a cognitive impairment [8]. However, use of RCD may also have some disadvantages. The quality and comprehensiveness of RCD may not be up to research standards, given that they were collected for other purposes than research [9]. For example, the preferred method for ascertaining falls is to use fall diaries with monthly reporting, which are only used in research cohorts [10]. In clinical practice, falls can be defined and determined inconsistently between healthcare professionals, and falls that did not require medical attention will often go unreported. Previous reviews have summarised the predictive performance of tools for estimating the fall risk, including prediction models [11–14]. However, they primarily examined models based on cohort data, omitting those derived from RCD. Comparing models derived from cohorts to those derived from RCD could offer valuable insights into their respective merits for fall prediction.

The aim of this review is to give an overview of prediction models for falls in community-dwelling older adults and to compare models derived from RCD against models derived from cohorts in terms of development, final included predictors, predictive performance, predicted outcomes, presentation, internal and external validation, reporting quality and risk of bias.

## Methods

This systematic review was reported according to the preferred reporting items for systematic reviews and meta-analyses statement (PRISMA) [15], and included in Supplementary Appendix 1. The review protocol was registered prospectively and published in the PROSPERO International Prospective Register of systematic reviews, registration number CRD42021240209.

### Literature search

A search was performed in Medline and Embase through Ovid, from inception until 1 January 2023, to identify studies reporting on the development and/or validation of models predicting falls in older adults in community-dwelling settings. The search contained the following key search concepts: ‘fall’, ‘aged’ and ‘prediction’. The first two search concepts (i.e. ‘fall’, ‘aged’) were adapted from the search filter previously created by a clinical librarian to identify studies about medication-related falls in older adults [16]. We further added prediction-related terms obtained from three experts in prediction models (AAH, MH, MS) to the filter including, among others, ‘area under the curve’, ‘discrimination’, ‘calibration’ and ‘sensitivity and specificity’ (see Supplementary Appendix 2 for the search strategy). Reference lists of two previous reviews [13, 14] and the included studies were subsequently searched to identify additional studies.

Studies were de-duplicated and two reviewers (BL, ND) independently screened retrieved studies for eligibility on title and abstract. Disagreements were resolved by discussion between the two reviewers. After consensus, full texts were assessed for eligibility by the reviewers (BL, ND). In case of doubt, a third reviewer (AAH) was involved to make the final decision.

### Selection criteria

Eligible studies needed to report on the development and/or external validation of one or more multivariable prediction models that include at least two predictors that provide an individual risk estimation (i.e. probabilistic or time-to-event models) for future falls, including injurious and recurrent falls. We only included studies where the sample consisted of community-dwelling older adults (e.g. not residing in rehabilitation centres), aged ≥60 years, or from which the majority were aged >65 years, or the mean age was >65 years. We excluded (1) studies pertaining to specific subpopulations (e.g. patients with Parkinson’s disease), (2) studies only aimed at identifying potential predictors for falls and studies that investigated the predictive potential of a single marker or test, (3) studies in which predictor weights were based on literature or clinical expertise, and (4) non-original research (e.g. reviews) and non-English studies.

### Data extraction

Data extraction was performed in accordance with the criteria of the checklist for critical appraisal and data extraction for systematic reviews of prediction modelling studies (CHARMS) checklist [17] (see Supplementary Appendix 3). Two reviewers (BL, ND) independently performed the data extraction on all these domains, where available.

### Risk of bias and quality assessment

We used the Prediction study Risk Of Bias Assessment Tool (PROBAST) [18] to evaluate risk of bias and concerns about applicability for models presented as part of the primary analysis. We used the transparent reporting of a multivariable prediction model for individual prognosis or diagnosis (TRIPOD) statement to assess adherence to reporting standards of prediction models [5]. Two reviewers (BL, ND) independently assessed the risk of bias and the reporting quality of each included study. Disagreements were resolved in consensus meetings and disagreements were resolved by involving two additional reviewers (AAH, MH).

### Summary measures and synthesis of results

We analysed individual models from studies, excluding those presented in sensitivity or additional analyses. Results were summarised using percentages, ranges, medians and interquartile ranges (IQR), alongside a narrative synthesis. We compared overall results and categorised them by data source (cohort-based or RCD-based) for direct comparison.

## Results

### Study selection

**Figure 1:**
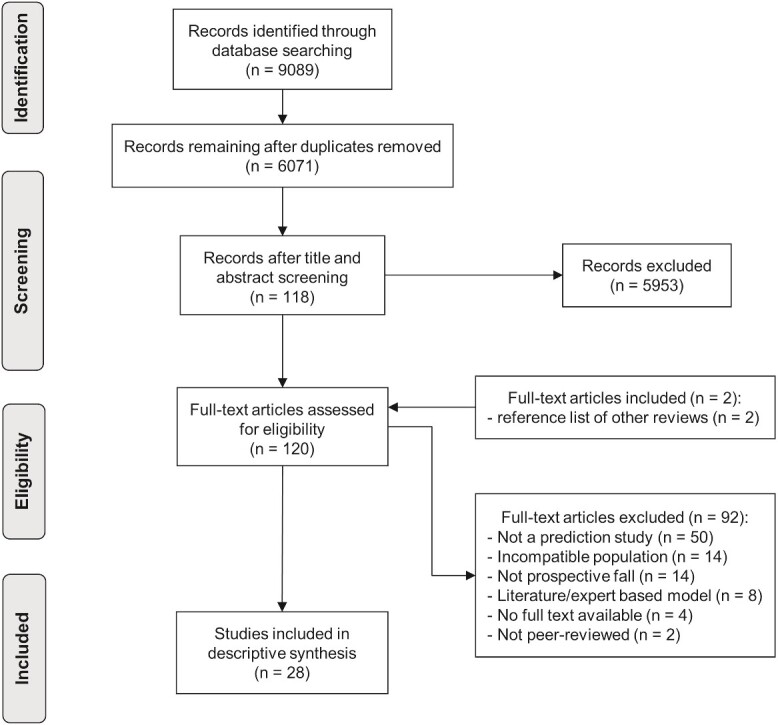
PRISMA flow diagram of study selection process.

The search retrieved 6071 studies after de-duplication. Title and abstract screening excluded 5953 studies and full-text screening excluded a further 92 studies (Figure 1). The list of the excluded articles with the reason of exclusion is available in Supplementary Appendix 4. After screening the reference list of other reviews, two studies were added. In total, 28 studies met the eligibility criteria and were included.

### Characteristics of included studies and participants

Table 1 summarises the characteristics of the included studies (see Supplementary Appendix 5 for details). Of the 28 included studies, 26 studies concerned the development of one or more prediction models [19, 21–33, 35–40, 42–47]. Two studies described the external validation of existing models, of which one was cohort-based and the other was RCD-based [20, 34]. We identified 30 prediction models, of which 23 [26–33, 35–40, 42–47] were cohort-based and seven [19, 21–25] were RCD-based. Cohort-based studies were conducted between 2000 and 2022, whereas RCD-based studies were conducted between 2014 and 2022. The median sample size of the cohort-based studies was 1365 (IQR 426–2766) individuals with a median age of 75 years (IQR 71–77). For the RCD-based studies, the median sample size was 90 441 (IQR 56 442–128 157) individuals with a median age of 74 years (IQR 72–75).

**Table 1:** Characteristics of the included studies

Study	Age^a^	Outcome and prediction horizon	*n* (outcome; %)^b^	# Predictors (# in final model)	Performance^c^
Dormosh *et al*. (2021) [19]	72 [68–78]	1-Year fall	36 470 (4778; 13.1)	79 (10)	AUC = 0.71 (IQR 0.70–0.71). PRAUC = 0.29 (IQR 0.28–0.30), calibration = ‘reasonable’ (plot)
Dormosh *et al*. (2022) [20]^d^	73 [69–79]	1-Year fall	38 133 (5124; 13.4)	NA	AUC = 0.69 (0.69–0.70), calibration = ‘reasonable’ (plot), calibration-in-the-large = 0.01 (−0.02 to 0.04), calibration slope = 0.88 (0.86–0.92)
Homer *et al*. (2017) [21]	75 [63–87]	2-Year fall	120 881 (12 431; 10.3)	62 (39)	AUC = 0.71 (CIs = NR), MAE = 0.8%
Oshiro *et al*. (2019) [22]	70 (8)	1-Year fall	90 441 (3415; 3.8)	45 (13)	AUC = 0.72 (CIs = NR), calibration = NR
Rafiq *et al*. (2014) [23]	74 [65–104]	30-Month fall or fracture	135 433 (10 766; 7.9)	33 (**Model 1:** 18; **Model 2:** 4)	**Model 1:** AUC = 0.70 (CIs = NR), calibration = ‘good’ (Hosmer–Lemeshow test) **Model 2:** AUC = NR, calibration = NR
Smith *et al*. (2016) [24]	NR	1-Year fall or fracture	74 751 (4941; 6.6)	116 (29)	AUC = 0.87 (CIs = NR), calibration = good (Hosmer–Lemeshow test)
Ye *et al*. (2020) [25]	75 (12)	1-Year fall	265 225 (4361; 1.6)	10 198 (NR)	AUC = 0.81 (CIs = NR), calibration = NR
Bath *et al*. (2000) [26]	NR	4-Year fall	435 (114; 26.2)	253 (16)	AUC = NR, calibration = NR
Bongue *et al*. (2011) [27]	71 (5)	1-Year fall	1759 (563; 32.0)	22 (6)	AUC = 0.70 (0.67–0.73), calibration = NR
Cella *et al*. (2020) [28]	77 (7)	1-Year fall	96 (32; 33.3)	56 (28)	AUC = 0.81 (0.72–0.90), calibration = NR
Coll-Planas *et al*. (2006) [29]	82 (SD = NR)	1-Year fall	192 (116; 60.4)	20 (2)	AUC = NR, calibration = NR
Covinsky *et al*. (2001) [30]	82 (4)	1-Year fall	557 (121; 21.7)	16 (3)	AUC = 0.70 (CIs = NR), calibration = NR
Delbaere *et al*. (2006) [31]	72 (6)	1-Year fall	257 (86; 33.5)	27 (2)	NR
Deschamps *et al*. (2016) [32]	70 (3)	1-Year fall	426 (82; 19.2)	73 (16)	AUC = 0.72 (CIs = NR), calibration = NR
Ek *et al*. (2019) [33]	73 (SD = NR)	5-Year first-time injurious fall	2808 (390; 13.9)	26 (6)	AUC for women = 0.75, for men = 0.77 (CIs = NR), calibration = NR
Frisendahl *et al*. (2020) [34]^d^	71 (SD = NR)	5-Year first-time injurious fall	2766 (177; 6.4)	NA	**Validation set 1:** AUC for women and men = 0.73 and 0.74 (CIs = NR), calibration = ‘good’ (Hosmer–Lemeshow test) **Validation set 2:** AUC for women and men = 0.73 and 0.74 (CIs = NR), calibration = ‘good’ (Hosmer–Lemeshow test)
Gade *et al*. (2021) [35]	82 [80–86]	1-Year number of falls	241 (87; 36.1)	34 (7)	AUC = NR, MAE = 0.88 (0.71–1.16)
Gadkaree *et al*. (2015) [36]	NR	**Model 1:** 1-year fall **Model 2:** 1-year recurrent falls	7609 (**Model 1** 2028, 26.7) (**Model 2** 957, 12.6)	15 (**Model 1:** 5; **Model 2:** 5)	**Model 1:** AUC = 0.70 (0.67–0.73), calibration = NR **Model 2:** AUC = 0.75 (0.73–0.80), calibration = NR
Ikeda *et al*. (2022) [37]	73 (SD = NR)	1-Year recurrent falls	61 885 (3359; 5.4)	142 (14)	AUC = 0.88 (CIs not = NR), calibration = NR
Kang *et al*. (2018) [38]	67 (6)	1-Year fall	619 (125; 20.2)	31 (5)	AUC = 0.75 (0.70–0.80), calibration = NR
Van de Loo *et al*. (2022) [39]	74 [69, 79]	**Model 1:** 1-year fall **Model 2:** recurrent falls	5722 (**Model 1:** 1868, 34.7) (**Model 2:** 702, 13.8)	82 (**Model 1:** 12; **Model 2:** 10)	**Model 1:** AUC = 0.65, calibration = ‘fair’ (plot) **Model 2:** AUC = 0.70, calibration = ‘fair’ (plot)
Makino *et al*. (2021) [40]	71 (5)	2-Year fall	2520 (415; 16.5)	9 (6)	AUC = 0.70 (0.68–0.72), calibration = NR
Okochi *et al*. (2006) [41]	76 (7)	6-Month fall	1378 (208; 15.1)	22 (5)	AUC = 0.74 (0.69–0.79), calibration = NR
Pluijm *et al*. (2006) [42]	75 (6)	3-Year recurrent falls	1365 (337; 24.7)	38 (10)	AUC = 0.71 (CIs = NR), calibration = ‘model fits data well’ (Hosmer–Lemeshow test)
Stalenhoef *et al*. (2002) [43]	78 (SD = NR)	3-Year recurrent falls	311 (81; 26.0)	36 (6)	AUC = 0.79 (CIs = NR), calibration = ‘good fit’ (Hosmer–Lemeshow test)
Stel *et al*. (2003) [44]	75 (6)	3-Year recurrent falls	1365 (337; 24.7)	34 (10)	NR
Tromp *et al*. (2001) [45]	75 (7)	**Model 1:** 1-year fall **Model 2:** 1-year recurrent falls	1285 (**Model 1:** 428, 33.3) (**Model 2:** 146, 11.4)	30 (**Model 1:** 4; **Model 2:** 4)	**Model 1:** AUC = 0.65 (CIs = NR), calibration = NR **Model 2:** AUC = 0.71, calibration = NR
Woo *et al*. (2009) [46]	NR	1-Year recurrent falls	3890 (391; 10.1)	49 (13)	AUC men and women = 0.75 (0.71–0.79) and 0.73 (0.69–0.76), calibration = NR

AUC, area under the receiver operating characteristic curve; CI, confidence interval; MAE, mean absolute error; NA, not applicable; NR, not reported; PRAUC, area under the precision recall curve; IQR, interquartile range.

^a^Data are reported as mean (SD) or median [IQR].

^b^In cases in which the outcome was the number of falls, we reported the number and percentage of participants who endured at least one fall. We estimated the number of events for studies in which only the percentage of events across the sample was reported.

^c^In cases in which the model was both validated on the basis of internal validation and apparent performance, only results from internal validation were reported. Apparent performance measures are reported for models that were not validated internally or externally. In the case of split-sample validation, only performance measures from the test set are reported. Values in parentheses represent 95% CIs unless stated otherwise. Other performance measures are given in Supplementary Appendix 5.

^d^External validation study.

### Outcome

For cohort-based models, predicted outcomes included any fall or time to any fall (13 models) [26–32, 36, 38–40, 45, 47], recurrent falls (8 models) [36, 37, 39, 42–46], injurious falls (1 model) [33] and number of falls (1 model) [35]. RCD-based models were developed to predict any fall (four models) [19, 21, 22, 25] and the composite ‘fall or fracture’ (three models) [23, 24]. Fall rate ranged from 5.4% to 60.4% for the cohort-based models and 1.6% to 13.1% for RCD-based models.

### Model development

Supplementary Appendix 6 summarises the modelling strategies used in the model development studies. Cohort-based models were mostly developed using logistic regression (13 models) [29–31, 36, 39, 42, 43, 45–47] and Cox proportional-hazards regression (3 models) [27, 33, 38]. RCD-based models mainly utilised penalised logistic regression with the least absolute shrinkage and selection operator (3 models) [19, 21, 22].

### Model performance and validation

Supplementary Appendix 7 summarises the model performance of the cohort-based and RCD-based models. Model discrimination in terms of the area under the receiver operating characteristic curve (AUC) (or equivalently the *c*-statistic) was reported for 18 cohort-based models [27, 28, 30, 32, 33, 36–40, 42, 43, 45–47] and calibration was reported for 5 cohort-based models [35, 39, 42, 43]. In comparison, discrimination measures were reported for six RCD-based models [19, 21–25] and calibration was reported for four models [19, 21, 23, 24]. Of the 23 cohort-based models, 12 models were not internally or externally validated [27, 29–32, 38, 42–46]; 1 model was only validated externally (using an independent sample) [33, 34]; and 10 models were only internally validated [28, 32, 36, 37, 39, 40, 47]. Of the seven RCD-based models, three models were not validated [23, 24], three models were validated only internally [21, 22, 25], and one model was validated both internally and externally [19, 20]. For one RCD model, it was unclear whether internal validation was performed [26]. AUCs ranged from 0.65 to 0.88 (median 0.72; IQR 0.65–0.77) for cohort-based models that were validated internally versus 0.71 to 0.81 (median 0.72; IQR 0.71–0.74) for RCD-based models.

With respect to external validation, two models (one cohort-based and one RCD-based) were externally validated. The cohort-based model of Ek *et al*. was externally validated on men and women in two independent cohort-based validation sets [33]. The AUCs were 0.73 for men and 0.74 for women in the first validation set and 0.72 for men and 0.89 for women in the second validation set, but a summary AUC value was not reported [34]. In terms of the RCD studies, the AUC of the model described by Dormosh *et al*. was 0.69 [95% confidence interval (CI) 0.69–0.70] when externally validated using an independent sample [20]. Both cohort-based and RCD-based models for which calibration was assessed reported satisfactory model calibration (i.e. ‘good’, ‘appropriate’, ‘reasonable’ or other synonyms).

### Predictors in the final model and model presentation

**Figure 2:**
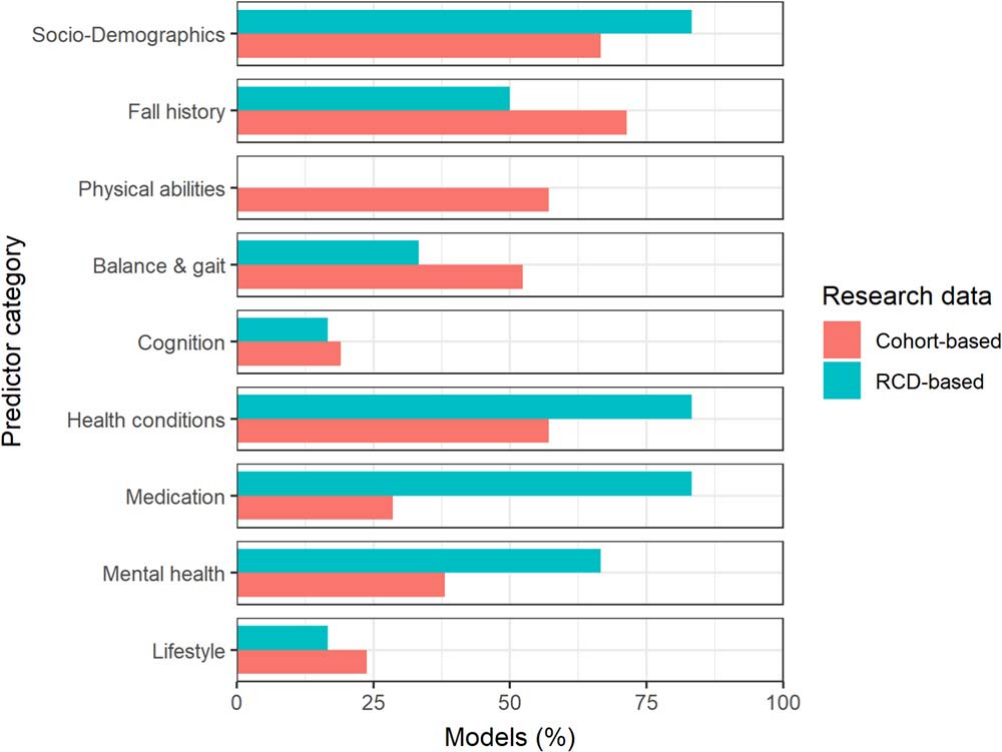
Overview of the proportion of final models containing specific category of predictors.

Cohort-based final models used a median of 6 predictors (IQR 5–11), while RCD-based models used a median of 16 predictors (IQR 11–26). One RCD-based model did not report the predictors in the final model [25]. The top five predictor categories found in cohort-based models were fall history, socio-demographics, health conditions, physical abilities, and balance and gait measures (Figure 2). In contrast, predictor categories in RCD-based models were socio-demographics, health conditions, medication, mental health and fall history (Figure 2). Supplementary Appendices 8 and 9 provide an overview of individual predictors in the final models and model presentation.

### Risk of bias

**Figure 3:**
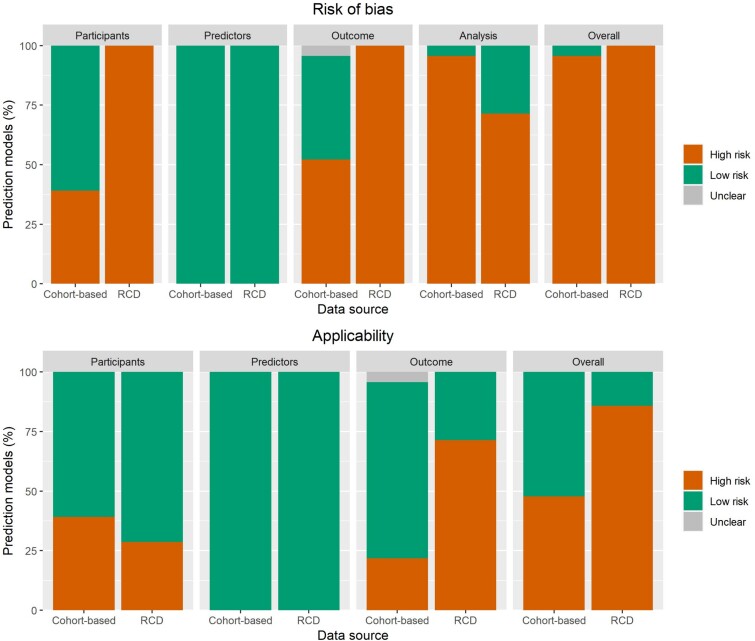
Risk of bias and applicability of the included developed models.

**Figure 4:**
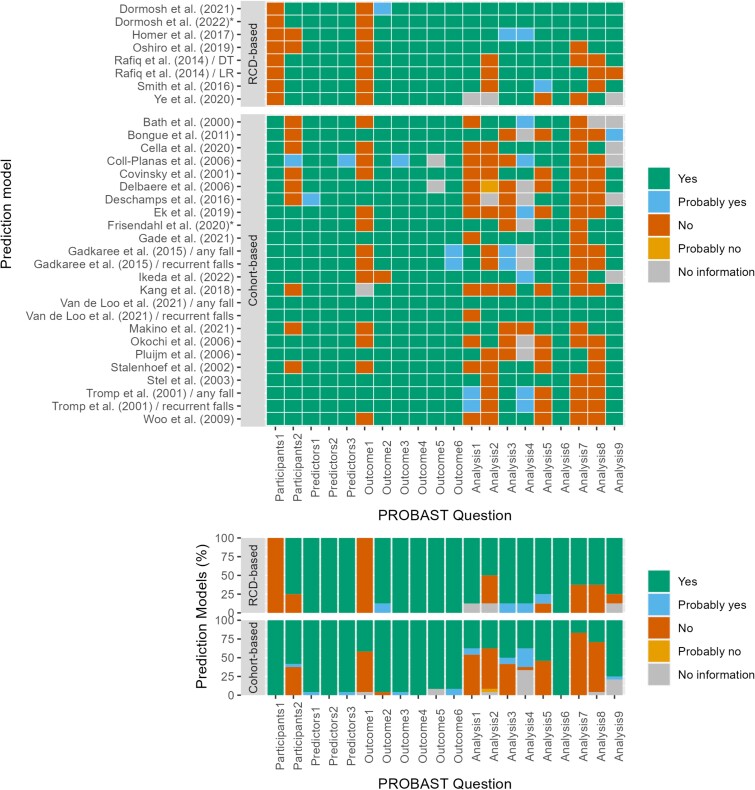
Heatmap of the scored signalling questions of the risk-of-bias assessment PROBAST. ^*^External validation study of one model.

Figure 3 compares bias and applicability ratings of cohort-based and RCD-based models. Out of 23 cohort-based models, only one had an overall low risk of bias to predict future falls [39]. All RCD-based models demonstrated an overall high risk of bias in at least one domain (Figure 4). Among the cohort-based models, nine had a high risk of bias due to the restrictive inclusion of relatively older people with lower fall risk [26–28, 30–32, 38, 40, 43]. In addition, we observed high risk of bias in the Outcome domain for 12 cohort-based models [26, 28–30, 33, 34, 36, 37, 40, 43, 46, 47], mainly due to falls not being ascertained using the recommended method of using daily calendars with monthly reporting [10]. As to the Analysis domain, all but one of the 23 cohort-based models [39] were found to have a high risk of bias. The main reasons for a high risk of bias score in this domain were lack of assessment of calibration (19 models) and/or discrimination (5 models) [26–38, 40, 44–47], inadequate internal validation (16 models) [27, 29–33, 36, 38, 42–47] and unnecessary dichotomisation of continuous variables (14 models) [25–29, 37–40, 43, 45, 47]. On the other hand, all RCD-based models had a high risk of bias in the Participants and Outcome domains due to the use of RCD and inappropriate fall determination. Compared with cohort-based models, RCD-based models scored better in the Analysis domain, although five of the seven had a risk of bias in at least one of the items (five models). This was mainly due to model calibration not being evaluated (three models) [22, 23, 25], inadequate internal validation (three models) [23, 24] and unnecessary dichotomisation of continuous variables (three models) [23, 24]. Regarding applicability, 12 cohort-based models scored as having a high concern of inapplicability to our research question [26, 27, 30–34, 38, 40, 43, 46, 47], as a result of selective sampling of participants with low fall risk (10 models) [26, 27, 30–34, 38, 40, 43] or deficiency in the determination of falls (5 models) [26, 30, 43, 46, 47]. High concern of inapplicability was scored for six RCD-based models [21–25], which was mainly due to how the outcome was determined and defined (five models) [22–25], as falls may have not entirely occurred in the community (i.e. in-hospital falls), or were defined to include fractures as well. Another reason was the presence of some participants who were likely to be institutionalised (two models), thereby rendering the models not entirely generalisable to community-dwelling older adults [21, 22].

### Quality of reporting

The median checklist adherence to TRIPOD was 66% (range: 50%–89%) for cohort-based studies and 71% (range: 55%–96%) for RCD-based studies. Supplementary Appendix 10 contains the scoring for each included study. Supplementary Appendices 11 and 12 provide the assessment of adherence to TRIPOD guidelines across sections and items.

## Discussion

### Principal findings

In this study, we identified 23 cohort-based and 7 RCD-based prediction models for falls in community-dwelling older people. Only two models (one cohort-based and one RCD-based) were externally validated. Most cohort-based and RCD-based models were assessed for model discrimination. Discrimination performance for internal validation was comparable for cohort-based and RCD-based models, with AUCs ranging from 0.65 to 0.88 versus 0.71 to 0.81, respectively. Model calibration was not assessed in most cohort-based studies, whereas most RCD-based models were assessed for model calibration. Most of the cohort-based models and all the RCD-based models demonstrated high risk of bias, primarily attributed to deficiencies in statistical analysis and fall determination, as well as inherent limitations associated with the use of RCD.

The majority of the cohort-based models demonstrated a high risk of bias, mostly due to narrow inclusion criteria, inappropriate determination of the outcome and deficiencies in the statistical analysis. These findings are in line with that of a previous systematic review which exclusively looked at cohort-based models [14]. Models that were based on cohort studies with narrow inclusion criteria may show limited performance in unrepresented patient groups, particularly frailer older adults with an increased risk of falls. In studies where the outcome was ascertained or defined inappropriately, the resultant model may systematically over- or underestimate the fall risk when applied to new patients. The majority of the cohort-based models were found to have an overall high risk of bias owing to limitations in the statistical analysis. In particular, omission of appropriate validation efforts, lack of calibration measures, inappropriate handling of missing data and unnecessary dichotomisation of continuous variables were frequent sources of bias. Failure to internally validate a fall prediction model can lead to overoptimistic estimates of its performance, which may not hold up when applied to new populations [48]. Calibration refers to the agreement between the actual and the predicted risks, an important aspect when employing model outputs for decision-making purposes [49]. The lack of calibration assessment can result in models that overestimate or underestimate the risk of falling, leading to misguided preventive interventions [49]. To prevent loss of information and to obtain unbiased estimates of the model’s performance, it is important to not categorise continuous variables and to use appropriate methods for handling missing data (e.g. multiple imputation) [18]. On the other hand, the high risk of bias in the RCD-based models is mainly attributable to the inherent nature of RCD and does not entirely reflect deficiencies in the applied methodologies. According to PROBAST, routinely collected data are not designed primarily for research purposes, and hence may be of lower quality and should be rated with high risk of bias as default.

Both cohort-based models and RCD-based models suffered from high risk of bias in fall ascertainment. More than half of the cohort-based models were developed using outcome data derived from questionnaires, which is not the recommended approach to ascertain falls due to recall bias concerns [50, 51]. Indeed, in cohort studies the incidence of any fall ranged from 5.4% to 60.4%, indicating some studies underestimated or overestimated the outcome, which can be partially attributed to recall bias. The preferred method for ascertaining falls is to use fall diaries with monthly reporting as advocated by the Prevention of Falls Network Europe [10]. Falls in routinely collected databases are encoded or registered in narrative texts. However, in clinical practice, fall incidents that do not require medical attention are usually underrepresented in RCD. Our results showed that the fall prevalence in RCD-based studies was noticeably lower than in cohort-based studies (cohort-based: 5.4%–60.4%; RCD-based: 1.6%–13.1%), indicating that falls were underreported. This is in line with previous work that found that non-injurious falls often go unreported to healthcare services [52].

Currently used fall-risk-assessment tools like the Berg Balance Scale and Timed Up and Go test have low predictive performance [13]. This poor performance may lead to unnecessary preventive measures or missed opportunities for intervention in those at higher fall risk. Given the multifactorial nature of falls, tools that cover a wider range of risk factors (e.g. impaired mobility, fall history, medications) may perform better. However, despite utilising various predictors, the discriminative performance of both cohort-based and RCD-based models was low to moderate. Use of additional candidate predictors in the modelling process may help to increase the predictive performance, assuming sufficient sample size. Particularly, environmental factors were overlooked as predictors in the development of fall prediction models, and medications were underutilised in cohort-based models.

The predictors in a prediction model significantly affect its performance. However, comparing the retained predictors in both the cohort-based and RCD-based models for a fair comparison is difficult because various factors impact the selection of the predictors (e.g. variable selection method, sample size, outcome rate and case mix). We noticed some overlap between the predictors retained in the final models of both cohort-based and RCD-based models. However, predictors related to physical abilities or balance and gait measures are more prevalent in cohort-based models as they barely exist in routinely collected data. Conversely, RCD-based models tend to have a large proportion of predictors related to socio-demographic information, medication and health conditions since these are abundant in RCD (e.g. EHR). An advantage of RCD-based predictors is their routine collection, eliminating the need for data collection or specialised assessments, as opposed to cohort-based predictors like gait speed. Nevertheless, it is important to interpret the predictors in fall prediction models with caution as some predictors may not have a direct causal relationship with the falls, but instead act as proxies for other unmeasured variables.

Some cohort-based studies [35, 39, 40] and RCD-based studies [19, 20] had good reporting quality, adhering to over 80% of TRIPOD criteria. Nevertheless, poor reporting was found in several areas, regardless of the data source used. Notably, about half of the included studies failed to report on model discrimination, calibration, internal validation or missing data, and failed to present their models in full with intercept or baseline risk accompanied with an explanation on using the model. Consequently, many of the included models are not available in a format such that they can be used, reproduced and critically evaluated, which hinders their translation into clinical practice [5]. This trend of suboptimal reporting has also been observed in several other systematic reviews on falls [14, 53, 54] and other domains [55–57].

### Strengths and limitations

This review’s strengths lie in its prespecified protocol registration and adherence to rigorous methodologies outlined by the CHARMS, TRIPOD, PROBAST and PRISMA guidelines.

There are limitations to our systematic review. First, our search was restricted to the Medline and Embase databases and we only included articles written in English. Second, the terminology used in articles related to fall prediction is not always consistent in the literature, which might have caused us to miss some articles. Third, our review solely encompassed fall-risk models that utilised probabilistic approaches, while we excluded other tools that classified patients into risk categories (i.e. low and high), such as the STEADI algorithm [58] and the World Falls Guidelines algorithm [4]. While such algorithms are simple to use, this often comes at the cost of dichotomising continuous predictors, potentially reducing their accuracy [59]. Finally, although the common adoption of an age cut-off at 65 for defining older adults aligns with existing literature, it may have inadvertently excluded potentially informative fall prediction models aimed at relatively younger populations.

### Clinical implications

Our results highlight different advantages and disadvantages of using either RCD or cohort data. RCD-based models may provide better coverage of patient groups that are difficult to enrol in cohort-based studies. However, they are likely unsuitable for predicting non-injurious falls, as our results suggest that these are underrepresented in routinely collected databases. This means that RCD-based models may help direct attention towards preventing the falls that are most severe. Nonetheless, even when non-injurious, a fall may still increase the risk of functional decline and other adverse health outcomes [2]. Therefore, when implementing a prediction model for falls, one should consider whether the goal is to address all falls or the most severe ones.

The discriminative performance of some prediction models was promising, with some reporting AUC values of >0.70 based on internal and external validation procedures. However, at the time of writing, none of the identified models can currently be recommended for clinical practice, given their identified high risk of bias and/or lack of external validation. Nonetheless, the models may be considered for clinicians’ use after rigorous external validation and impact studies. External validation is necessary to ensure the model’s generalisability in other independent populations [5, 60]. An impact study is required to assess how use of the model impacts clinical decision making and health outcomes in patients [61].

### Recommendations for researchers

Given the methodological shortcomings mentioned earlier, we provide some recommendations for researchers to improve the overall quality of prediction modelling studies in fall research. First, researchers are encouraged to follow the rigorous methods set forth by Steyerberg and Vergouwe [62, 63] for model development and validation, to avoid common methodological pitfalls when developing prediction models for falls. Second, researchers should consider externally validating and updating existing models as opposed to developing new ones. Finally, researchers are advised to adhere to the TRIPOD guidelines when reporting prediction models for falls.

## Conclusion

While there are many prediction models derived from data of prospective research cohorts for falls in community-dwelling older people, those derived from RCD are still in early stages but rapidly growing, and provide comparable predictive performance with no additional data collection efforts. All but one cohort-based model had high risk of bias, primarily due to deficiencies in statistical analysis and outcome determination. Future studies should focus on methodological and reporting quality to improve the quality of fall prediction models.

## Supplementary Material

aa-23-1460-File006_afae131

## Notes

Noman Dormosh and Bob van de Loo: shared first co-authors.
