# Supplementary material for: A systematic review of fall prediction models for community-dwelling older adults: comparison between models based on research cohorts and models based on routinely collected data
Source: Age Ageing. 2024 Jul 9;53(7):afae131. doi: 10.1093/ageing/afae131 (PMC11231951; doi:10.1093/ageing/afae131)
Supplement: aa-23-1460-File006_afae131 [file aa-23-1460-file006_afae131.docx]

**A systematic review of fall prediction models for community-dwelling older adults: comparison between models based on research cohorts and models based on routinely-collected data**

**Appendices**

[Appendix 1: Preferred Reporting Items for Systematic Reviews and Meta-Analyses (PRISMA) checklist 2](#_Toc167096091)

[Appendix 2: Search strategy in Medline and Embase through Ovid 4](#_Toc167096092)

[Appendix 3: Data items used to describe the characteristics of the included studies 5](#_Toc167096093)

[Appendix 4: Studies excluded during full-text screening 7](#_Toc167096094)

[Appendix 5: Characteristics of the Included Studies 13](#_Toc167096095)

[Appendix 6: Type of modelling strategy used 18](#_Toc167096096)

[Appendix 7: Model performance and validation of cohort-based and RCD-based models 19](#_Toc167096097)

[Appendix 8: Predictors in the final models and their respective proportions 20](#_Toc167096098)

[Appendix 9: Model presentation of the included fall prediction models 23](#_Toc167096099)

[Appendix 10: Overall adherence to the TRIPOD per study 24](#_Toc167096100)

[Appendix 11: Proportion of studies that adhered to the TRIPOD per section 26](#_Toc167096101)

[Appendix 12: Adherence to the TRIPOD checklist across the items 27](#_Toc167096102)

[References (for appendix) 28](#_Toc167096103)

# Appendix 1: Preferred Reporting Items for Systematic Reviews and Meta-Analyses (PRISMA) checklist

| **Section and Topic** | **Item #** | **Checklist item** | **Location where item is reported** |
| --- | --- | --- | --- |
| **TITLE** | | |  |
| Title | 1 | Identify the report as a systematic review. | Title page |
| **ABSTRACT** | | |  |
| Abstract | 2 | See the PRISMA 2020 for Abstracts checklist. | Title page |
| **INTRODUCTION** | | |  |
| Rationale | 3 | Describe the rationale for the review in the context of existing knowledge. | 1. |
| Objectives | 4 | Provide an explicit statement of the objective(s) or question(s) the review addresses. | 1. |
| **METHODS** | | |  |
| Eligibility criteria | 5 | Specify the inclusion and exclusion criteria for the review and how studies were grouped for the syntheses. | 2.1 |
| Information sources | 6 | Specify all databases, registers, websites, organisations, reference lists and other sources searched or consulted to identify studies. Specify the date when each source was last searched or consulted. | 2.1 |
| Search strategy | 7 | Present the full search strategies for all databases, registers and websites, including any filters and limits used. | 2.1 & Appendix 2 |
| Selection process | 8 | Specify the methods used to decide whether a study met the inclusion criteria of the review, including how many reviewers screened each record and each report retrieved, whether they worked independently, and if applicable, details of automation tools used in the process. | 2.1 |
| Data collection process | 9 | Specify the methods used to collect data from reports, including how many reviewers collected data from each report, whether they worked independently, any processes for obtaining or confirming data from study investigators, and if applicable, details of automation tools used in the process. | 2.1 & 2.2 |
| Data items | 10a | List and define all outcomes for which data were sought. Specify whether all results that were compatible with each outcome domain in each study were sought (e.g. for all measures, time points, analyses), and if not, the methods used to decide which results to collect. | 2.3 & Appendix 3 |
|  | 10b | List and define all other variables for which data were sought (e.g. participant and intervention characteristics, funding sources). Describe any assumptions made about any missing or unclear information. | 2.3 & Appendix 3 |
| Study risk of bias assessment | 11 | Specify the methods used to assess risk of bias in the included studies, including details of the tool(s) used, how many reviewers assessed each study and whether they worked independently, and if applicable, details of automation tools used in the process. | 2.4 |
| Effect measures | 12 | Specify for each outcome the effect measure(s) (e.g. risk ratio, mean difference) used in the synthesis or presentation of results. | NA |
| Synthesis methods | 13a | Describe the processes used to decide which studies were eligible for each synthesis (e.g. tabulating the study intervention characteristics and comparing against the planned groups for each synthesis (item #5)). | 2.5 |
|  | 13b | Describe any methods required to prepare the data for presentation or synthesis, such as handling of missing summary statistics, or data conversions. | NA |
|  | 13c | Describe any methods used to tabulate or visually display results of individual studies and syntheses. | NA |
|  | 13d | Describe any methods used to synthesize results and provide a rationale for the choice(s). If meta-analysis was performed, describe the model(s), method(s) to identify the presence and extent of statistical heterogeneity, and software package(s) used. | NA |
|  | 13e | Describe any methods used to explore possible causes of heterogeneity among study results (e.g. subgroup analysis, meta-regression). | NA |
|  | 13f | Describe any sensitivity analyses conducted to assess robustness of the synthesized results. | NA |
| Reporting bias assessment | 14 | Describe any methods used to assess risk of bias due to missing results in a synthesis (arising from reporting biases). | NA |
| Certainty assessment | 15 | Describe any methods used to assess certainty (or confidence) in the body of evidence for an outcome. | NA |
| **RESULTS** | | |  |
| Study selection | 16a | Describe the results of the search and selection process, from the number of records identified in the search to the number of studies included in the review, ideally using a flow diagram. | 3.1 & Fig 1 |
|  | 16b | Cite studies that might appear to meet the inclusion criteria, but which were excluded, and explain why they were excluded. | Appendix 4 |
| Study characteristics | 17 | Cite each included study and present its characteristics. | 3.2 – 3.8 & Table 1 |
| Risk of bias in studies | 18 | Present assessments of risk of bias for each included study. | 3.9 & Fig 3, 4 |
| Results of individual studies | 19 | For all outcomes, present, for each study: (a) summary statistics for each group (where appropriate) and (b) an effect estimate and its precision (e.g. confidence/credible interval), ideally using structured tables or plots. | 3.9 & Fig 3, 4 |
| Results of syntheses | 20a | For each synthesis, briefly summarise the characteristics and risk of bias among contributing studies. | NA |
|  | 20b | Present results of all statistical syntheses conducted. If meta-analysis was done, present for each the summary estimate and its precision (e.g. confidence/credible interval) and measures of statistical heterogeneity. If comparing groups, describe the direction of the effect. | NA |
|  | 20c | Present results of all investigations of possible causes of heterogeneity among study results. | NA |
|  | 20d | Present results of all sensitivity analyses conducted to assess the robustness of the synthesized results. | NA |
| Reporting biases | 21 | Present assessments of risk of bias due to missing results (arising from reporting biases) for each synthesis assessed. | NA |
| Certainty of evidence | 22 | Present assessments of certainty (or confidence) in the body of evidence for each outcome assessed. | NA |
| **DISCUSSION** | | |  |
| Discussion | 23a | Provide a general interpretation of the results in the context of other evidence. | 4.1 & 4.3 & 5 |
|  | 23b | Discuss any limitations of the evidence included in the review. | 4.2 |
|  | 23c | Discuss any limitations of the review processes used. | 4.2 |
|  | 23d | Discuss implications of the results for practice, policy, and future research. | 5 |
| **OTHER INFORMATION** | | |  |
| Registration and protocol | 24a | Provide registration information for the review, including register name and registration number, or state that the review was not registered. | 2. |
|  | 24b | Indicate where the review protocol can be accessed, or state that a protocol was not prepared. | 2. |
|  | 24c | Describe and explain any amendments to information provided at registration or in the protocol. | NA |
| Support | 25 | Describe sources of financial or non-financial support for the review, and the role of the funders or sponsors in the review. | Title page |
| Competing interests | 26 | Declare any competing interests of review authors. | Title page |
| Availability of data, code and other materials | 27 | Report which of the following are publicly available and where they can be found: template data collection forms; data extracted from included studies; data used for all analyses; analytic code; any other materials used in the review. | NA |

# Appendix 2: Search strategy in Medline and Embase through Ovid

| 1. accidental falls/ 2. falling/ 3. (fall? or fell or falling or fallen or faller or fallen or stumble? or stumbling or stumbles or slip or slips or slipping or slipped or trip or tripped).ti,ab,kw,kf. 4. 1 or 2 or 3 5. Geriatric assessment/ or aging/ or frail elderly/ or exp aged/ or middle aged/ 6. exp aging/ or exp aged/ or elderly care/ or geriatric care/ 7. ((old* adj3 person?) or (old* adj3 patient?) or (old* adj3 client?) or (old* adj3 adult) or (older adj3 m?n) or (older adj3 wom?n) or senior? or elder* or aged or geriatric* or gerontolog* or frailty or aging or ageing or postmenopausal women or geriatric assessment).ti,ab,kw,kf. 8. 5 or 6 or 7 9. (predict* model* or prognostic model* or AUC or area under the curve or receiver operating characteristic or ROC or discriminat* or calibration or brier score or (sensitivity adj5 specificity) or (observed adj3 expected) or predictive value or goodness of fit or c-index or c-statistic or hosmer-lemeshow or machine learning or (predict* adj3 performance)).ab,kw,ti,kf. 10. 4 and 8 and 9 |
| --- |

# Appendix 3: Data items used to describe the characteristics of the included studies

| **Publication** | Author & publication date |
| --- | --- |
| **Objective** | Primary study objective [Prediction model development (D) or validation (V) study] |
| **Source of data** | Source of data (e.g. cohort, case-control, registry data, etc) |
| **Participants** | Participant eligibility and recruitment method (e.g. consecutive participants, location, number of centres, in or exclusion criteria) |
|  | Number of participants |
|  | Participant description |
|  | Study dates |
| **Outcomes to be predicted** | Definition and method for measurement of outcome |
|  | The same definition used in all patients? |
|  | The outcome assessment without knowledge of candidate predictors? |
|  | Type of outcome (e.g., single or combined endpoints) |
|  | Time of outcome occurrence or summary of duration of follow-up |
| **Candidate predictors** | Type and number of predictors |
|  | Definition and method for measurement of candidate predictors |
|  | Timing of predictor assessment |
|  | Were predictors assessed blinded for outcome? |
|  | Handling of predictors in the modelling (e.g. continuous, linear, non-linear transformations, etc.) |
| **Sample size** | Number of participants |
|  | Number of events |
|  | Number of events in relation to candidate predictors (EPV) |
| **Missing data** | Number of participants with any missing value |
|  | Number of participants with missing data for each predictor |
|  | Handling of missing data (e.g. complete-cases, imputation) |
| **Model development** | Modelling method |
|  | Modelling assumptions satisfied |
|  | Method for selection of predictors for inclusion in multivariable modelling |
|  | Method for selection of predictors during multivariable modelling (e.g. forward, backward, etc) and criteria used (e.g. p-value, Akaike Information Criterion) |
|  | Shrinkage of predictor weight or regression coefficients (e.g., no shrinkage, uniform shrinkage, penalized estimation) |
| **Model performance** | Calibration and discrimination measures |
|  | Classification measures |
| **Model evaluation** | Method used for testing model performance: development dataset only or separate external volition |
|  | In case of poor validation, whether model was adjusted or updated |
| **Results** | Final multivariable models presented, including predictor weights, intercept, baseline survival, model performance |
|  | Any alternative presentation (e.g. sum score, nomogram) |
|  | Comparison of the distribution of predictors (including missing data) for development and validation datasets |
| **Interpretation and discussion** | Interpretation of presented models |
|  | Comparison with other studies, discussion, generalizability |

# Appendix 4: Studies excluded during full-text screening

| **Title** | **Reason of exclusion** | **Authors** | **Journal** | **Volume** | **Issue** |
| --- | --- | --- | --- | --- | --- |
| Local dynamic stability during gait for predicting falls in elderly people: A one-year prospective study | Not a prediction study | Bizovska, L. and Svoboda, Z. and Janura, M. and Bisi, M. C. and Vuillerme, N. | PLoS ONE | 13 |  |
| The predictive value of gait speed and maximum step length for falling in community-dwelling older persons | Not a prediction study | Bongers, K. T. J. and Schoon, Y. and Graauwmans, M. J. and Schers, H. J. and Melis, R. J. and Olde rikkert, M. G. M. | Age and Ageing | 44 | 2 |
| FRAT-up, a Web-based fall-risk assessment tool for elderly people living in the community | Literature/expert based model | Cattelani, L. and Palumbo, P. and Palmerini, L. and B and inelli, S. and Becker, C. and Chesani, F. and Chiari, L. | Journal of medical Internet research | 17 | 2 |
| Development of a fall-risk assessment profile for community-dwelling older adults by using the National Health Interview Survey in Taiwan | Not prospective fall | Chen, P. L. and Lin, H. Y. and Ong, J. R. and Ma, H. P. | BMC public health | 20 | 1 |
| Validation of a fall-risk screening test, the Elderly Fall Screening Test (EFST), for community-dwelling elderly | Literature/expert based model | Cwikel, J. G. and Fried, A. V. and Biderman, A. and Galinsky, D. | Disability and Rehabilitation | 20 | 5 |
| Slow Processing Speed Predicts Falls in Older Adults With a Falls History: 1-Year Prospective Cohort Study | Not a prediction study | Davis, J. C. and Best, J. R. and Khan, K. M. and Dian, L. and Lord, S. and Delbaere, K. and Hsu, C. L. and Cheung, W. and Chan, W. and Liu-Ambrose, T. | Journal of the American Geriatrics Society | 65 | 5 |
| The harmonic ratio of trunk acceleration predicts falling among older people: results of a 1-year prospective study | Not a prediction study | Doi, T. and Hirata, S. and Ono, R. and Tsutsumimoto, K. and Misu, S. and Ando, H. | Journal of neuroengineering and rehabilitation | 10 |  |
| Using random forest plots to examine predictive and discriminative ability of mobility measures for future falls | Not a prediction study | Donoghue, O. and Hern and ez, B. and O'Connell, M. and Kenny, R. A. | Age and Ageing. Conference: 67th Annual and Scientific Meeting of the Irish Gerontological Society, Innovation, Advances and Excellence in Ageing. Ireland. | 48 |  |
| Faller Classification in Older Adults Using Wearable Sensors Based on Turn and Straight-Walking Accelerometer-Based Features | Not a prediction study | Drover, D. and Howcroft, J. and Kofman, J. and Lemaire, E. D. | Sensors | 17 | 6 |
| Falls risk assessment score (FRAS): Time to rethink | Incompatible population | El Miedany, Y. and El Gaafary, M. and Toth, M. and Palmer, D. and Ahmed, I. | Journal of Clinical Gerontology and Geriatrics | 2 | 1 |
| New neural network classifier of fall-risk based on the Mahalanobis distance and kinematic parameters assessed by a wearable device | Not prospective fall | Giansanti, D. and Macellari, V. and Maccioni, G. | Physiological Measurement | 29 | 3 |
| Using supervised learning machine algorithm to identify future fallers based on gait patterns: A two-year longitudinal study | Incompatible population | Gillain, S. and Boutaayamou, M. and Schwartz, C. and Bruls, O. and Bruyere, O. and Croisier, J. L. and Salmon, E. and Reginster, J. Y. and Garraux, G. and Petermans, J. | Experimental Gerontology | 127 | 110730 |
| Evaluation of falls risk in community-dwelling older adults using body-worn sensors | Not a prediction study | Greene, B. R. and Doheny, E. P. and Walsh, C. and Cunningham, C. and Crosby, L. and Kenny, R. A. | Gerontology | 58 | 5 |
| Quantitative falls risk assessment using the timed up and go test | Not prospective fall | Greene, B. R. and Odonovan, A. and Romero-Ortuno, R. and Cogan, L. and Scanaill, C. N. and Kenny, R. A. | IEEE Transactions on Biomedical Engineering | 57 | 12 |
| Failure of falls risk screening tools to predict outcome: A prospective cohort study | Incompatible population | Harper, K. J. and Barton, A. D. and Arendts, G. and Edwards, D. G. and Petta, A. C. and Celenza, A. | Emergency Medicine Journal | 35 | 1 |
| A modified fall risk assessment tool that is specific to physical function predicts falls in community-dwelling elderly people | Literature/expert based model | Hirase, T. and Inokuchi, S. and Matsusaka, N. and Nakahara, K. and Okita, M. | Journal of geriatric physical therapy (2001) | 37 | 4 |
| A frailty profile for use in the community: Inclusion of psychological variables improves prediction of care needs and falls | No full text available | Holl and , C. and Garner, I. and O'Donnell, J. and Gwyther, H. | Age and Ageing | 48 |  |
| Prospective Fall-Risk Prediction Models for Older Adults Based on Wearable Sensors | Not a prediction study | Howcroft, J. and Kofman, J. and Lemaire, E. D. | IEEE Transactions on Neural Systems and Rehabilitation Engineering | 25 | 10 |
| Prospective elderly fall prediction by older-adult fall-risk modeling with feature selection | Not a prediction study | Howcroft, J. and Lemaire, E. D. and Kofman, J. | Biomedical Signal Processing and Control | 43 |  |
| Elderly fall risk prediction using static posturography | Not a prediction study | Howcroft, J. and Lemaire, E. D. and Kofman, J. and McIlroy, W. E. | PLoS ONE | 12 |  |
| Improved prediction of falls in community-dwelling older adults through phase-dependent entropy of daily-life walking | Not a prediction study | Ihlen, E. A. F. and van Schooten, K. S. and Bruijn, S. M. and van Dieen, J. H. and Vereijken, B. and Helbostad, J. L. and Pijnappels, M. | Frontiers in Aging Neuroscience | 10 | 44 |
| Falling in the elderly: Do statistical models matter for performance criteria of fall prediction? Results from two large population-based studies | Not a prediction study | Kabeshova, A. and Launay, C. P. and Gromov, V. A. and Fantino, B. and Levinoff, E. J. and Allali, G. and Beauchet, O. | European Journal of Internal Medicine | 27 |  |
| Gait dynamics to optimize fall risk assessment in geriatric patients admitted to an outpatient diagnostic clinic | Not a prediction study | Kikkert, L. H. J. and De Groot, M. H. and Van Campen, J. P. and Beijnen, J. H. and Hortobagyi, T. and Vuillerme, N. and Lamoth, C. C. J. | PLoS ONE | 12 |  |
| Frailty predicts short-term incidence of future falls among British community-dwelling older people: a prospective cohort study nested within a randomised controlled trial | Not a prediction study | Kojima, G. and Kendrick, D. and Skelton, D. A. and Morris, R. W. and Gawler, S. and Iliffe, S. | BMC geriatrics | 15 |  |
| Development and validation of an algorithm to assess risk of first-time falling among home care clients | Incompatible population | Kuspinar, A. and Hirdes, J. P. and Berg, K. and McArthur, C. and Morris, J. N. | BMC geriatrics | 19 | 1 |
| Predicting falls within the elderly community: Comparison of postural sway, reaction time, the Berg balance scale and the Activities-specific Balance Confidence (ABC) scale for comparing fallers and non-fallers | Not prospective fall | Lajoie, Y. and Gallagher, S. P. | Archives of Gerontology and Geriatrics | 38 | 1 |
| The Clinical Potential of Frailty Indicators on Identifying Recurrent Fallers in the Community: The Mr. Os and Ms. OS Cohort Study in Hong Kong | Not a prediction study | Lam, F. M. H. and Leung, J. C. S. and Kwok, T. C. Y. | Journal of the American Medical Directors Association | 20 | 12 |
| Gait and patient characteristics that discriminate fallers from non-fallers in a geriatric population | Not a prediction study | Lamoth, C. and De Groot, M. and Van Campen, J. and Van Der Jagt, H. and Beijnen, J. H. | European Geriatric Medicine | 1 |  |
| Short-Physical Performance Battery (SPPB) score is associated with falls in older outpatients | Not prospective fall | Lauretani, F. and Ticinesi, A. and Gionti, L. and Prati, B. and Nouvenne, A. and Tana, C. and Meschi, T. and Maggio, M. | Aging Clinical and Experimental Research | 31 | 10 |
| Validation of the Saskatoon Falls Prevention Consortium's Falls Screening and Referral Algorithm | Not prospective fall | Lawson, S. N. and Zaluski, N. and Petrie, A. and Arnold, C. and Basran, J. and Dal Bello-Haas, V. | Physiotherapy Canada | 65 | 1 |
| Maximum step length as a potential screening tool for falls in non-disabled older adults living in the community | Not a prediction study | Lindemann, U. and Lundin-Olsson, L. and Hauer, K. and Wengert, M. and Becker, C. and Pfeiffer, K. | Aging Clinical and Experimental Research | 20 | 5 |
| Validation of an accelerometer-based fall prediction model | Not a prediction study | Liu, Y. and Redmond, S. J. and Shany, T. and Woolgar, J. and Narayanan, M. R. and Lord, S. R. and Lovell, N. H. | Conference proceedings : .. |  |  |
| External validation of a simple risk model to predict recurrent falling in older community-living people in primary care | Not peer-reviewed | Logghe, I. and Peeters, G. and Rademaker, A. and Bierma-Zeinstra, S. M. A. and Van Rossum, E. and Faber, M. and Koes, B. and Verhagen, A. P. | Physiotherapy (United Kingdom) | 1 |  |
| Simple physiological and clinical tests for the accurate prediction of falling in older people | No full text available | Lord, S. R. and Clark, R. D. | Gerontology | 42 | 4 |
| Validity of the Falls Risk for Older People in the Community (FROP-Com) tool to predict falls and fall injuries for older people presenting to the emergency department after falling | Incompatible population | Mascarenhas, M. and Hill, K. D. and Barker, A. and Burton, E. | European Journal of Ageing | 16 | 3 |
| Prediction of Falls Using a 3-m Zigzag Walk Test | Not a prediction study | Masuda, S. and Suganuma, K. and Kaneko, C. and Hoshina, K. and Suzuki, T. and Serita, T. and Sakakibara, R. | Journal of Physical Therapy Science | 25 | 9 |
| Validation of a risk stratification tool for fall-related injury in a state-wide cohort | Incompatible population | McCoy, T. H. and Castro, V. M. and Cagan, A. and Roberson, A. M. and Perlis, R. H. | BMJ Open | 7 |  |
| Obstacle course performance and risk of falling in community-dwelling elderly persons | Not a prediction study | Means, K. M. and Rodell, D. E. and O'Sullivan, P. S. | Archives of Physical Medicine and Rehabilitation | 79 | 12 |
| Do voluntary step reactions in dual task conditions have an added value over single task for fall prediction? A prospective study | Not a prediction study | Melzer, I. and Kurz, I. and Shahar, D. and Oddsson, L. I. E. | Aging Clinical and Experimental Research | 22 | 5 |
| Gait disturbances as specific predictive markers of the first fall onset in elderly people: A two-year prospective observational study | Not a prediction study | Mignardot, J. B. and Deschamps, T. and Barrey, E. and Auvinet, B. and Berrut, G. and Cornu, C. and Constans, T. and De Decker, L. | Frontiers in Aging Neuroscience | 6 |  |
| Activities-specific balance confidence scale for predicting future falls in Indian older adults | Not a prediction study | Moiz, J. A. and Bansal, V. and Noohu, M. M. and Gaur, S. N. and Hussain, M. E. and Anwer, S. and Alghadir, A. | Clinical Interventions in Aging | 12 |  |
| Development of a scale to identify the fall-prone patient | No full text available | Morse, J. M. and Morse, R. M. and Tylko, S. J. | Canadian Journal on Aging | 8 | 4 |
| Screening for falls in community-dwelling elderly | Not a prediction study | Murphy, M. A. and Olson, S. L. and Protas, E. J. and Overby, A. R. | Journal of Aging and Physical Activity | 11 | 1 |
| Deep Learning to Predict Falls in Older Adults Based on Daily-Life Trunk Accelerometry | Not a prediction study | Nait Aicha, A. and Englebienne, G. and van Schooten, K. S. and Pijnappels, M. and Krose, B. | Sensors | 18 | 5 |
| Development and preliminary examination of the predictive validity of the falls risk assessment tool (FRAT) for use in primary care | Literature/expert based model | N and y, S. and Parsons, S. and Cryer, C. and Underwood, M. and Rashbrook, E. and Carter, Y. and Eldridge, S. and Close, J. and Skelton, D. and Taylor, S. and Feder, G. | Journal of Public Health | 26 | 2 |
| Predictive Performance of a Fall Risk Assessment Tool for Community-Dwelling Older People (FRAT-up) in 4 European Cohorts | Literature/expert based model | Palumbo, P. and Klenk, J. and Cattelani, L. and B and inelli, S. and Ferrucci, L. and Rapp, K. and Chiari, L. and Rothenbacher, D. | Journal of the American Medical Directors Association | 17 | 12 |
| Fall risk assessment tools for elderly living in the community: Can we do better? | Not a prediction study | Palumbo, P. and Palmerini, L. and B and inelli, S. and Chiari, L. | PLoS ONE | 10 |  |
| Mobility assessment: Sensitivity and specificity of measurement sets in older adults | Not a prediction study | Panzer, V. P. and Wakefield, D. B. and Hall, C. B. and Wolfson, L. I. | Archives of Physical Medicine and Rehabilitation | 92 | 6 |
| Ability of Self-Reported Frailty Components to Predict Incident Disability, Falls, and All-Cause Mortality: Results From a Population-Based Study of Older British Men | Not a prediction study | Papachristou, E. and Wannamethee, S. G. and Lennon, L. T. and Papacosta, O. and Whincup, P. H. and Iliffe, S. and Ramsay, S. E. | Journal of the American Medical Directors Association | 18 | 2 |
| Does physical performance testing predict falls? Results from the hertfordshire cohort study | Not a prediction study | Parsons, C. and Edwards, M. H. and Cooper, C. and Dennison, E. M. and Ward, K. A. | Osteoporosis International | 28 |  |
| Using the Hendrich II Inpatient Fall Risk Screen to Predict Outpatient Falls After Emergency Department Visits | Incompatible population | Patterson, B. W. and Repplinger, M. D. and Pulia, M. S. and Batt, R. J. and Svenson, J. E. and Trinh, A. and Mendonca, E. A. and Smith, M. A. and Hamedani, A. G. and Shah, M. N. | Journal of the American Geriatrics Society | 66 | 4 |
| Training and interpreting machine learning algorithms to evaluate fall risk after emergency department visits | Incompatible population | Patterson, B. W. and Sah, V. and Smith, M. and Engstrom, C. and Mendonca, E. A. and Pulia, M. and Repplinger, M. and Hamedani, A. G. and Page, D. and Shah, M. and Kennedy, M. | Academic Emergency Medicine | 26 |  |
| Validation of the LASA fall risk profile for recurrent falling in older recent fallers | Incompatible population | Peeters, G. M. E. E. and Pluijm, S. M. F. and Van Schoor, N. M. and Elders, P. J. M. and Bouter, L. M. and Lips, P. | Journal of Clinical Epidemiology | 63 | 11 |
| A predictive model of isolated and recurrent falls in functionally independent community-dwelling older adults | Not a prediction study | Perez-Ros, P. and Martinez-Arnau, F. M. and Orti-Lucas, R. M. and Tarazona-Santabalbina, F. J. | Brazilian journal of physical therapy | 23 | 1 |
| Fall risk screening in the elderly: A comparison of the minimal chair height standing ability test and 5-repetition sit-to-stand test | Not prospective fall | Reider, N. and Gaul, C. | Archives of Gerontology and Geriatrics | 65 |  |
| Sensitivity and specificity of the minimal chair height standing ability test: a simple and affordable fall-risk screening instrument | Not prospective fall | Reider, N. C. and Naylor, P. J. and Gaul, C. | Journal of geriatric physical therapy (2001) | 38 | 2 |
| Predictors of falls among elderly people. Results of two population-based studies | Not a prediction study | Robbins, A. S. and Rubenstein, L. Z. and Josephson, K. R. and Schulman, B. L. and Osterweil, D. and Fine, G. | Archives of Internal Medicine | 149 | 7 |
| A two-question tool to assess the risk of repeated falls in the elderly | Not a prediction study | Rodriguez-Molinero, A. and Galvez-Barron, C. and Narvaiza, L. and Minarro, A. and Ruiz, J. and Valldosera, E. and Gonzalo, N. and Ng, T. and Sanguino, M. J. and Yuste, A. | PLoS ONE | 12 |  |
| The spatial parameters of gait and their association with falls, functional decline and death in older adults: a prospective study | Not a prediction study | Rodriguez-Molinero, A. and Herrero-Larrea, A. and Minarro, A. and Narvaiza, L. and Galvez-Barron, C. and Gonzalo Leon, N. and Valldosera, E. and de Mingo, E. and Macho, O. and Aivar, D. and Pinzon, E. and Alba, A. and Passarelli, J. and Stasi, N. and Valverde, R. A. and Kruse, L. and Felipe, E. and Collado, I. and Sabater, J. B. | Scientific Reports | 9 | 1 |
| The reliability and predictive accuracy of the falls risk for older people in the community assessment (FROP-Com) tool | Literature/expert based model | Russell, M. A. and Hill, K. D. and Blackberry, I. and Day, L. M. and Dharmage, S. C. | Age and Ageing | 37 | 6 |
| Fall predictors in the community dwelling elderly: A cross sectional and prospective cohort study | Not a prediction study | Sai, A. J. and Gallagher, J. C. and Smith, L. M. and Logsdon, S. | Journal of Musculoskeletal Neuronal Interactions | 10 | 2 |
| Fusion of Clinical, Self-Reported, and Multisensor Data for Predicting Falls | Not a prediction study | Silva, J. and Sousa, I. and Cardoso, J. S. | IEEE Journal of Biomedical and Health Informatics | 24 | 1 |
| Fall risk in Chinese community-dwelling older adults: A physiological profile assessment study | Not a prediction study | Siong, K. H. and Kwan, M. M. S. and Lord, S. R. and Lam, A. K. C. and Tsang, W. W. N. and Cheong, A. M. Y. | Geriatrics and Gerontology International | 16 | 2 |
| The construction of a patient record-based risk model for recurrent falls among elderly people living in the community | Not prospective fall | Stalenhoef, P. A. and Diederiks, J. P. M. and Knottnerus, J. A. and De Witte, L. P. and Crebolder, H. F. J. M. | Family Practice | 17 | 6 |
| Enhancing the ability of postural control analyses to differentiate between fallers and non-fallers: Scaling stance width data to body size | Not a prediction study | Swanenburg, J. and De Bruin, E. D. | Physiotherapy (United Kingdom) | 1 |  |
| Thai falls risk assessment test (Thai-FRAT) developed for community-dwelling thai elderly | Not prospective fall | Thiamwong, L. and Thamarpirat, J. and Maneesriwongul, W. and Jitapunkul, S. | Journal of the Medical Association of Thailand | 91 | 12 |
| The development and validation of a brief performance-based fall risk assessment tool for use in primary care | Literature/expert based model | Tiedemann, A. and Lord, S. R. and Sherrington, C. | The journals of gerontology |  | 8 |
| Identifying older people at high risk of future falls: Development and validation of a screening tool for use in emergency departments | Incompatible population | Tiedemann, A. and Sherrington, C. and Orr, T. and Hallen, J. and Lewis, D. and Kelly, A. and Vogler, C. and Lord, S. R. and Close, J. C. T. | Emergency Medicine Journal | 30 | 11 |
| The comparative ability of eight functional mobility tests for predicting falls in community-dwelling older people | Not a prediction study | Tiedemann, A. and Shimada, H. and Sherrington, C. and Murray, S. and Lord, S. | Age and Ageing | 37 | 4 |
| "Fall risk index" helps clinicians identify high-risk individuals | Literature/expert based model | Toba, K. and Kikuchi, R. and Iwata, A. and Kozaki, K. | Japan Medical Association Journal | 52 | 4 |
| Clinical utility of Hendrich II scores in predicting outpatient falls | Incompatible population | Trinh, A. and Patterson, B. W. and Shah, M. N. and Pulia, M. and Repplinger, M. D. | Academic Emergency Medicine | 25 |  |
| Ambulatory fall-risk assessment: amount and quality of daily-life gait predict falls in older adults | Not a prediction study | van Schooten, K. S. and Pijnappels, M. and Rispens, S. M. and Elders, P. J. and Lips, P. and van Dieen, J. H. | The journals of gerontology |  | 5 |
| Daily-life gait quality as predictor of falls in older people: A 1-year prospective cohort study | Not a prediction study | Van Schooten, K. S. and Pijnappels, M. and Rispens, S. M. and Elders, P. J. M. and Lips, P. and Daffertshofer, A. and Beek, P. J. and Van Dieen, J. H. | PLoS ONE | 11 |  |
| Pns199 Predicting Falls in Elderly Patients Using Claims Data: A Time-Aware Deep Learning Approach | No full text available | Wang, Y. and McClellan, M. and Kinsman, W. and Teigl and , C. and Kilgore, K. | Value in Health | 22 |  |
| Is the SPPB a useful tool for stratifying fall risk in primary care? | Not a prediction study | Welch, S. A. and Ward, R. E. and Beauchamp, M. K. and Leveille, S. G. and Bean, J. F. | Journal of the American Geriatrics Society | 67 |  |
| Predictive performance of four frailty measures in an older Australian population | Not a prediction study | Widagdo, I. S. and Pratt, N. and Russell, M. and Roughead, E. E. | Age and Ageing | 44 | 6 |
| Validating a fall risk assessment tool | Not prospective fall | Wong, W. P. and Yong, L. M. | Annals of the Academy of Medicine Singapore | 1 |  |
| Fall Risk Classification in Community-Dwelling Older Adults Using a Smart Wrist-Worn Device and the Resident Assessment Instrument-Home Care: Prospective Observational Study | Not a prediction study | Yang, Y. and Hirdes, J. P. and Dubin, J. A. and Lee, J. | 24058297 | 2 | 1 |
| Development and initial validation of the Falls Efficacy Scale-International (FES-I) | Not a prediction study | Yardley, L. and Beyer, N. and Hauer, K. and Kempen, G. and Piot-Ziegler, C. and Todd, C. | Age and Ageing | 34 | 6 |
| Validation of FallScreen to identify fall risk among older singaporeans | Not prospective fall | Yong, L. M. and Wong, W. P. | Proceedings of Singapore Healthcare | 2 |  |
| Predicting Falls Among Community-Dwelling Older Adults: A Demonstration of Applied Machine Learning | Not a prediction study | Yang, R., Plasek, J. M., Cummins, M. R., & Sward, K. A. | CIN: Computers, Informatics, Nursing | 39 | 5 |
| Serious Falls in Middle-Aged Veterans: Development and Validation of a Predictive Risk Model | Incompatible population | Serious Falls in Middle-Aged Veterans: Development and Validation of a Predictive Risk Model | Journal of the American Geriatrics Society | 68 | 12 |
| Deep Learning for Fall Risk Assessment with Inertial Sensors: Utilizing Domain Knowledge in Spatiooral Gait Parameters | Not prospective fall | Tunca, C., Salur, G., & Ersoy, C. | IEEE journal of biomedical and health informatics | 24 | 7 |
| Machine Learning in Aging: An Example of Developing Prediction Models for Serious Fall Injury in Older Adults | Not a prediction study | Speiser, J. L., Callahan, K. E., Houston, D. K., Fanning, J., Gill, T. M., Guralnik, J. M., ... & Miller, M. E. | The Journals of Gerontology: Series A | 76 | 4 |
| Two-item fall screening tool identifies older adults at increased risk of falling after emergency department visit | Incompatible population | Solie, C. J., Swanson, M. B., Harland, K., Blum, C., Kin, K., & Mohr, N. | Western journal of emergency medicine | 21 | 5 |
| Foreseeing future falls with accelerometer features in active community-dwelling older persons with no recent history of falls | Not a prediction study | Bet, P., Castro, P. C., & Ponti, M. A. | Experimental gerontology | 143 |  |
| Impact of Altering Data Granularity Levels on Predictive Modelling: A Case Study of Fall Risk Prediction in Older Persons | Not a prediction study | Dormosh, N., Abu-Hanna, A., van der Velde, N., & Schut, M. C. | MIE |  |  |
| A Postural Assessment Utilizing Machine Learning Prospectively Identifies Older Adults at a High Risk of Falling | Not a prediction study | Forth, K. E., Wirfel, K. L., Adams, S. D., Rianon, N. J., Lieberman-Aiden, E., & Madansingh, S. I. | Frontiers in medicine | 7 |  |
| Using pharmacy dispensing data to predict falls in older individuals | Not a prediction study | Gemmeke, M., Koster, E. S., Pajouheshnia, R., Kruijtbosch, M., Taxis, K., & Bouvy, M. L. | British Journal of Clinical Pharmacology | 87 | 3 |
| Development and external validation of a risk prediction model for falls in patients with an indication for antihypertensive treatment: retrospective cohort study. | Incompatible population | Archer, L., Koshiaris, C., Lay-Flurrie, S., Snell, K. I., Riley, R. D., Stevens, R., ... & Sheppard, J. P. | BMJ | 379 |  |
| External validation and further exploration of fall prediction models based on questionnaires and daily-life trunk accelerometry. | Not peer-reviewed | Zhang, Y., Weijer, R., van Schooten, K. S., Bruijn, S. M., & Pijnappels, M. | bioRxiv |  |  |
| Development and validation of a continuous fall risk score in community-dwelling older people: an ecological approach. | Not prospective fall | Bravo, J., Rosado, H., Tomas-Carus, P., Carrasco, C., Batalha, N., Folgado, H., & Pereira, C. | BMC public health | 21 | 2 |

# Appendix 5: Characteristics of the Included Studies

| Study | Age^a^ | Outcome and prediction horizon | *n* (outcome; %)^b^ | # Predictors (# in final model) | Modelling method (presentation) | Type of validation | Performance^c^ |
| --- | --- | --- | --- | --- | --- | --- | --- |
| Dormosh et al. (2021) [1] | 72 [68-78] | 1-year fall | 36,470 (4,778; 13.1) | 79 (10) | LASSO LR (regression formula) | CV | AUC = 0.71 (IQR 0.70–0.71). PRAUC = 0.29 (IQR 0.28–0.30), calibration = ‘reasonable’ (plot), Brier score = 0.11, sens = 0.62, spec = 0.70, PPV = 0.24 |
| Dormosh et al. (2022) [2] | 73 [69-79] | 1-year fall | 38,133 (5,124; 13.4) | NA | NA | External | AUC = 0.69 (0.69-0.70), calibration = ‘reasonable’ (plot), calibration-in-the-large = 0.01 (-0.02-0.04), calibration slope = 0.88 (0.86-0.92) |
| Homer et al. (2017) [3] | 75 [63-87] | 2-year fall | 120,881 (12,431; 10.3) | 62 (39) | LASSO LR (regression formula) | Split-sample | AUC = 0.71 (CIs = NR), MAE = 0.8% |
| Oshiro et al. (2019) [4] | 70 (8) | 1-year fall | 90,441 (3,415; 3.8) | 45 (13) | LASSO LR (regression formula) | Split-sample | AUC = 0.72 (CIs = NR), calibration = NR, sens = 0.73, spec = 0.70, PPV = 0.09, NPV = 0.99 |
| Rafiq et al. (2014) [5] | 74 [65-104] | 30-month fall or fracture | 135,433 (10,766; 7.9) | 33 (**Model 1:** 18; **Model 2:** 4) | **Model 1:** LR (score chart) **Model 2:** DT (regression tree) | NR | **Model 1:**  AUC = 0.70 (CIs = NR), calibration = ‘good' (Hosmer-Lemeshow test), sens = 0.68, spec = 0.60, PPV = 0.13, NPV = 0.96 **Model 2:** AUC = NR, calibration = NR, sens = 0.53, spec = 0.71, PPV = 0.14 |
| Smith et al. (2016) [6] | NR | 1-year fall or fracture | 74,751 (4,941; 6.6) | 116 (29) | Multilevel LR (regression formula) | NR | AUC = 0.87 (CIs = NR), calibration = good (Hosmer-Lemeshow test), sens = 0.81, spec = 0.78, NPV = 0.98, PPV = 0.21 |
| Ye et al. (2020) [7] | 75 (12) | 1-year fall | 265,225 (4,361; 1.6) | 10,198 (NR) | XGBoost (model not reported) | Split-sample | AUC = 0.81 (CIs = NR), calibration = NR |
| Bath et al. (2000) [8] | NR | 4-year fall | 435 (114; 26.2) | 253 (16) | Genetic Algorithm Neural Network (model not reported) | NR | AUC= NR, calibration = NR, sens = 0.31, PPV = 0.57, spec = 0.92, NPV = 0.79 |
| Bongue et al. (2011) [9] | 71 (5) | 1-year fall | 1,759 (563; 32.0) | 22 (6) | Cox regression (score chart) | NR | AUC = 0.70 (0.67–0.73), calibration = NR, sens = 0.70, spec = 0.60, PPV = 0.46, NPV = 0.81 |
| Cella et al. (2020) [10] | 77 (7) | 1-year fall | 96 (32; 33.3) | 56 (28) | LASSO LR (model not reported) | CV | AUC = 0.81 (0.72–0.90), calibration = NR, sens = 0.78, spec = 0.74, PPV = 0.69 |
| Coll-Planas et al. (2006) [11] | 82 (SD = NR) | 1-year fall | 192 (116; 60.4) | 20 (2) | LR (score chart) | NR | AUC = NR, calibration = NR, sens = 0.85, spec = 0.42, PPV = 0.69, NPV = 0.65 |
| Covinsky et al. (2001) [12] | 82 (4) | 1-year fall | 557 (121; 21.7) | 16 (3) | LR (score chart) | NR | AUC = 0.70 (CIs = NR), calibration = NR |
| Delbaere et al. (2006) [13] | 72 (6) | 1-year fall | 257 (86; 33.5) | 27 (2) | LR (regression formula) | NR | NR |
| Deschamps et al. (2016) [14] | 70 (3) | 1-year fall | 426 (82; 19.2) | 73 (16) | Decision tree (regression tree) | Split-sample | AUC = 0.72 (CIs = NR), calibration = NR |
| Ek et al. (2019) [15] | 73 (SD = NR) | 5-year first-time injurious fall | 2,808 (390; 13.9) | 26 (6) | Cox regression (score chart) | NR | AUC for women = 0.75, for men = 0.77 CIs = NR), calibration = NR, sens for women and men = 0.69 and 0.70, spec for women and men = 0.72 and 0.71 |
| Frisendahl et al. (2020) [16] | 71 (SD = NR) | 5-year first-time injurious fall | 2,766 (177; 6.4) | NA | NA | External | **Validation set 1:** AUC for women and men = 0.73 and 0.74 (CIs = NR), calibration = 'good' (Hosmer-Lemeshow test), sens = 0.64 and spec = 0.69 for women, sens = 0.68 and spec = 0.69 for men  **Validation set 2:**  AUC for women and men = 0.73 and 0.74 (CIs = NR), calibration = 'good' (Hosmer-Lemeshow test), sens = 0.64 and spec = 0.74 for women, sens = 0.94 and spec = 0.68 for men |
| Gade et al. (2021) [17] | 82 [80-86] | 1-year number of falls | 241 (87; 36.1) | 34 (7) | LASSO Poisson regression (regression formula) | Bootstrap sampling | AUC = NR, MAE = 0.88 (0.71–1.16) |
| Gadkaree et al. (2015) [18] | NR | **Model 1:** 1-year fall **Model 2:** 1-year recurrent falls | 7,609 (**Model 1**: 2,028, 26.7) (**Model 2**: 957, 12.6) | 15 (**Model 1:** 5; **Model 2:** 5) | LR (regression formula) | Split-sample | **Model 1:** AUC = 0.70 (0.67-0.73), calibration = NR **Model 2:**  AUC = 0.75 (0.73-0.80), calibration = NR |
| Ikeda et al. (2022)[19] | 73 (SD = NR) | 1-year recurrent falls | 61,885 (3,359; 5.4) | 142 (14) | XGBoost (model not reported) | CV | AUC = 0.88 (CIs not= NR), calibration = NR, accuracy = 0.88, F1 score = 0.89 |
| Kang et al. (2018) [20] | 67 (6) | 1-year fall | 619 (125; 20.2) | 31 (5) | Cox regression (score chart) | NR | AUC = 0.75 (0.70-0.80). calibration = NR, sens = 0.71, spec = 0.70, PPV = 0.38, NPV = 0.91 |
| Van de Loo et al. (2022) [21] | 74 [69, 79] | **Model 1:** 1-year fall  **Model 2:** recurrent falls | 5722 (**Model 1:** 1,868, 34.7)  (**Model 2:** 702 , 13.8) | 82 (**Model 1:** 12; **Model 2:** 10) | LR (regression formula) | Internal-external CV | **Model 1:**  AUC = 0.65, calibration = ‘fair’ (plot) **Model 2:**  AUC = 0.70, calibration = ‘fair’ (plot) |
| Makino et al. (2021) [22] | 71 (5) | 2-year fall | 2,520 (415; 16.5) | 9 (6) | DT (regression tree) | CV | AUC = 0.70 (0.68−0.72), calibration = NR, accuracy = 0.65, sens = 0.62, spec = 0.69, PPV = 0.66, NPV = 0.64 |
| Okochi et al. (2006) [23] | 76 (7) | 6-month fall | 1,378 (208; 15.1) | 22 (5) | LR (score chart) | Split-sample | AUC = 0.74 (0.69-0.79), calibration = NR |
| Pluijm et al. (2006) [24] | 75 (6) | 3-year recurrent falls | 1,365 (337; 24.7) | 38 (10) | LR (score chart) | NR | AUC = 0.71 (CIs = NR), calibration = ‘model fits data well’ (Hosmer-Lemeshow test), sens = 0.59, spec = 0.71 |
| Stalenhoef et al. (2002) [25] | 78 (SD = NR) | 3-year recurrent falls | 311 (81; 26.0) | 36 (6) | LR (score chart) | NR | AUC = 0.79 (CIs = NR), calibration = ‘good fit’ (Hosmer-Lemeshow test), sens = 0.59, spec = 0.87 , PPV = 0.52, NPV = 0.90 |
| Stel et al. (2003) [26] | 75 (6) | 3-year recurrent falls | 1,365 (337; 24.7) | 34 (10) | Tree-structured survival analysis (regression tree) | NR | NR |
| Tromp et al. (2001) [27] | 75 (7) | **Model 1:** 1-year fall  **Model 2:** 1-year recurrent falls | 1,285 (**Model 1:** 428, 33.3)  (**Model 2:** 146, 11.4) | 30 (**Model 1:** 4; **Model 2:** 4) | LR (score chart) | NR | **Model 1:**  AUC = 0.65 (CIs = NR), calibration = NR  **Model 2:** AUC = 0.71, calibration = NR, sens = 0.54, spec 0.79, PPV = 0.25, NPV = 0.93 |
| Woo et al. (2009) [28] | NR | 1-year recurrent falls | 3,890 (391; 10.1) | 49 (13) | LR (score chart) | NR | AUC men and women = 0.75 (0.71-0.79) and 0.73 (0.69-0.76), calibration = NR, sens men and women = 0.65 and 0.64, spec men and women = 0.78 and 0.54 |
| AUC- area under the receiver operating characteristic curve; CI- confidence interval; CV- cross-validation; LASSO- least absolute shrinkage and selection operator; LR- logistic regression; MAE- mean absolute error; NA- not applicable; NPV- negative predictive values; NR- not reported; PPV- positive predictive values; PRAUC- area under the precision recall curve; IQR- interquartile range; sens- sensitivity; spec- specificity; DT- decision tree; XGBoost- eXtreme Gradient Boosting  ^a^data is reported as mean (standard deviation) or median [IQR]  ^b^In cases in which the outcome was the number of falls, we reported the number and percentage of participants who endured at least one fall. We estimated the number of events for studies in which only the percentage of events across the sample was reported.  ^c^In cases in which the model was both validated on the basis of internal validation and apparent performance, only results from internal validation were reported. Apparent performance measures are reported for models that were not validated internally or externally. In the case of split-sample validation, only performance measures from the test set are reported. Values in parentheses represent 95% CIs unless stated otherwise. | | | | | | | |

# Appendix 6: Type of modelling strategy used

| **Algorithm** | **Cohort-based models (n=23)** | **RCD-based models (n=7)** |
| --- | --- | --- |
| **Unregularised logistic regression** | 13 regression [11–13,18,21,24,25,27–29] | 1 [5] |
| **Cox regression** | 3 [9,15,20] | - |
| **Regularised logistic regression** | 1 [30] | 3 [1,3,4] |
| **Decision tree** | 2 [14,22] | 1 [5] |
| **eXtreme Gradient Boosting (XGBoost)** | 1 [19] | 1 [7] |
| **Regularised poisson regression** | 1 [17] | - |
| **Tree-structured survival analysis** | 1 [26] | - |
| **Genetic Algorithm Neural Network** | 1 [8] | - |
| **multilevel logistic regression** | - | 1 [6] |

# Appendix 7: Model performance and validation of cohort-based and RCD-based models

| **Algorithm** | **Cohort-based models (n=23)** | **RCD-based models (n=7)** |
| --- | --- | --- |
| Number of models assessed for model discrimination | 18 | 6 |
| Median AUC (IQR) for internally validated models | 0.72 (0.65-0.77) | 0.72 (0.71-0.74) |
| Number of models assessed for model calibration | 5 | 4 |
| Number of models validated |  | |
| Internally | 10 | 3 |
| Externally | 1 | 1 |
| Number of models not validated internally or externally | 12 | 3 |
| IQR- interquartile range | | |

# Appendix 8: Predictors in the final models and their respective proportions

| **Predictor / Category** | **Cohort-based studies (n=21)^a^** | **RCD-based studies (n=7)^a^** |
| --- | --- | --- |
| **Socio-demographics** | | |
| Age | 7 | 5 |
| Sex | 4 | 5 |
| Body mass index or lean mass | 2 | 0 |
| Body weight | 1 | 0 |
| Dogs or cats in household | 2 | 0 |
| Educational level | 4 | 0 |
| Attendance at social gatherings | 1 | 0 |
| Sufficient contact with friends or family | 1 | 0 |
| Preferred hand of respondent | 1 | 0 |
| Living alone | 2 | 0 |
| **Fall history** | | |
| Fall history | 15 | 3 |
| **Physical abilities** | | |
| Functional limitations (ADL / IADL) | 9 | 0 |
| Grip strength | 6 | 0 |
| Self-perceived health | 2 | 0 |
| Walking aid | 1 | 0 |
| Musculoskeletal functional limitations | 1 | 0 |
| Receiving help | 1 | 0 |
| Previous injury | 0 | 1 |
| Self-reported decline in walking speed | 1 | 0 |
| Back bended | 1 | 0 |
| Flexibility | 1 | 0 |
| **Balance and gait** | | |
| Balance measures or self-perceived balance problems | 6 | 1 |
| Gait measure or self-perceived gait problems | 2 | 0 |
| Gait. balance and/or strength combined | 5 | 1 |
| **Cognition** | | |
| Visual impairment | 4 | 1 |
| Hearing impairment | 1 | 0 |
| Doman specific tests | 1 | 0 |
| **Health conditions** | | |
| Rheumatic disease | 3 | 4 |
| Dizziness | 4 | 1 |
| Incontinence (bowel or urine) | 3 | 3 |
| Diabetes | 2 | 1 |
| Cardiovascular disease | 0 | 3 |
| Others | 2 | 3 |
| Lung disease | 0 | 1 |
| Parkinson's disease | 0 | 1 |
| Dementia | 0 | 1 |
| Malaise and fatigue | 0 | 1 |
| Lower limb pain | 1 | 0 |
| Headaches | 1 | 0 |
| Central Nervous System Infection | 0 | 1 |
| Hereditary And Degenerative Nervous System Conditions | 0 | 1 |
| Urinary tract disease | 0 | 1 |
| Nocturia | 0 | 1 |
| Polycythaemia | 0 | 1 |
| Anaemia | 0 | 1 |
| Chronic kidney disease | 0 | 1 |
| Ankle hypoesthesia | 1 | 0 |
| Foot pathology | 1 | 0 |
| Fracture | 0 | 1 |
| Pain | 1 | 0 |
| Comorbidity index | 0 | 1 |
| **Medication** | | |
| Number of medications or specific drugs | 3 | 2 |
| Hypnotics or sedatives | 1 | 1 |
| Psychotropics | 1 | 1 |
| Antiepileptics | 1 | 1 |
| Antihypertensives | 0 | 2 |
| Opioids | 0 | 2 |
| Antidepressants | 0 | 2 |
| Laxatives | 0 | 1 |
| Antiadrenergic Agents | 0 | 1 |
| Diuretics | 0 | 1 |
| Vasodilators | 0 | 1 |
| Muscle Relaxants | 0 | 1 |
| Other Analgesics And Antipyretics | 0 | 1 |
| Dopaminergic Agents | 0 | 1 |
| Anxiolytics | 0 | 1 |
| Psychostimulants. agents used for ADHD and Nootropics | 0 | 1 |
| Anti-Dementia drugs | 0 | 1 |
| Drugs used for peptic ulcer and GERD | 0 | 1 |
| Drugs for urinary frequency and incontinence | 1 | 0 |
| Proton pump inhibitors | 0 | 1 |
| Anticonvulsants | 0 | 1 |
| Other drugs | 0 | 2 |
| **Mental health** | | |
| Depression or depressive symptoms | 6 | 4 |
| Concerns about falling | 3 | 0 |
| Sleep quality | 1 | 1 |
| Other mental health disorders | 0 | 2 |
| Memory and concentration problems | 0 | 1 |
| Anxiety | 0 | 1 |
| Delirium | 0 | 1 |
| Schizophrenia And Other Psychotic Disorders | 0 | 1 |
| Feeling full of energy | 1 | 0 |
| **Lifestyle** | | |
| Alcohol consumption | 3 | 0 |
| Physical activity | 2 | 0 |
| Smoking | 1 | 1 |
| Others | 1 | 0 |
| **Others** | | |
| Systolic blood pressure | 1 | 0 |
| Whole milk consumption | 1 | 0 |
| Nutritional score | 1 | 0 |
| Choking | 1 | 0 |
| Difficulties in eating tough foods | 1 | 0 |
| AE investigation | 0 | 1 |
| Self-perceived falls risk | 1 | 0 |
| Inpatient/outpatient episode | 0 | 1 |
| Inpatient/outpatient visit | 0 | 1 |
| Admission to hospital | 0 | 1 |
| Dry mouth | 1 | 0 |
| Number of remaining teeth | 1 | 0 |
| Sense of coherence | 1 | 0 |
| ^a^Numbers represent the number of cohort- or RCD-based studies in which the predictor was included in one or more final models | | |

# Appendix 9: Model presentation of the included fall prediction models

| **Presentation** | **Cohort-based studies (n=23)** | **RCD-based studies (n=7)** |
| --- | --- | --- |
| **Score chart** | 11 (48%) [9,11,12,15,20,24,25,27–29] | 1 (14%) model [5] |
| **Regression formula** | 6 (26%) [13,17,18,21] | 4 (57%) models [1,3,4,6] |
| **Regression tree** | 3 (13%) [14,22,26] | 1 (14%) [5] |
| **No model presentation** | 3 (13%) [8,19,30] | 1 (14%) [7] |

# Appendix 10: Overall adherence to the TRIPOD per study

| **Study** | **Type of prediction model study** | **TRIPOD Adherence Score (%)** |
| --- | --- | --- |
| **Studies that used routinely-collected data** | | |
| Dormosh et al., 2021 [1] | Development study | 93 |
| (Dormosh et al., 2022) [2] | Validation study | 96 |
| (Homer et al., 2017) [3] | Development study | 79 |
| (Oshiro et al., 2019) [4] | Development study | 63 |
| (Rafiq et al., 2014) [5] | Development study | 71 |
| (Smith et al., 2016) [6] | Development study | 70 |
| (Ye et al., 2020) [7] | Development study | 55 |
| **Studies that used data from research cohorts** | | |
| (Bath et al., 2000) [8] | Development study | 52 |
| (Bongue et al., 2011) [9] | Development study | 75 |
| (Cella et al., 2020) [10] | Development study | 77 |
| (Coll-Planas et al., 2006) [11] | Development study | 64 |
| (Covinsky et al., 2001) [12] | Development study | 61 |
| (Delbaere et al., 2006) [13] | Development study | 61 |
| (Deschamps et al., 2016) [14] | Development study | 59 |
| (Ek et al., 2019) [15] | Development study | 76 |
| (Frisendahl et al., 2020) [16] | Validation study | 70 |
| (Gade et al., 2021) [17] | Development study | 89 |
| (Gadkaree et al., 2015) [18] | Development study | 61 |
| (Ikeda et al., 2022) [31] | Development study | 56 |
| (Kang et al., 2018) [20] | Development study | 68 |
| (Van De Loo et al., 2022) [21] | Development study | 85 |
| (Makino et al., 2021) [22] | Development study | 82 |
| (Toba et al., 2007) [23] | Development study | 50 |
| (Pluijm et al., 2006) [24] | Development study | 79 |
| (Stalenhoef et al., 2002) [25] | Development study | 66 |
| (Stel et al., 2003) [26] | Development study | 64 |
| (Tromp et al., 2001) [27] | Development study | 68 |
| (Woo et al., 2009) [28] | Development study | 61 |

# Appendix 11: Proportion of studies that adhered to the TRIPOD per section


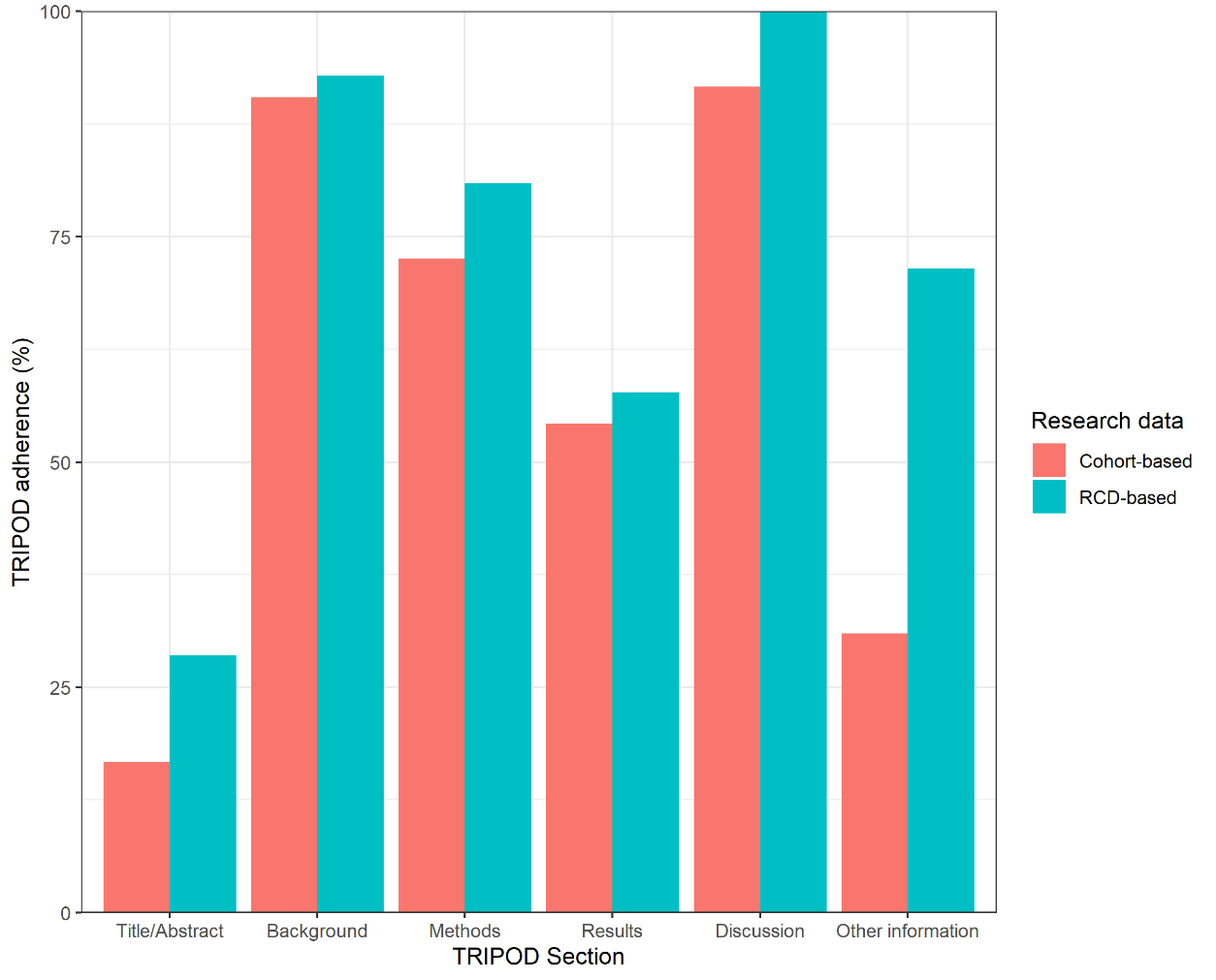


# Appendix 12: Adherence to the TRIPOD checklist across the items


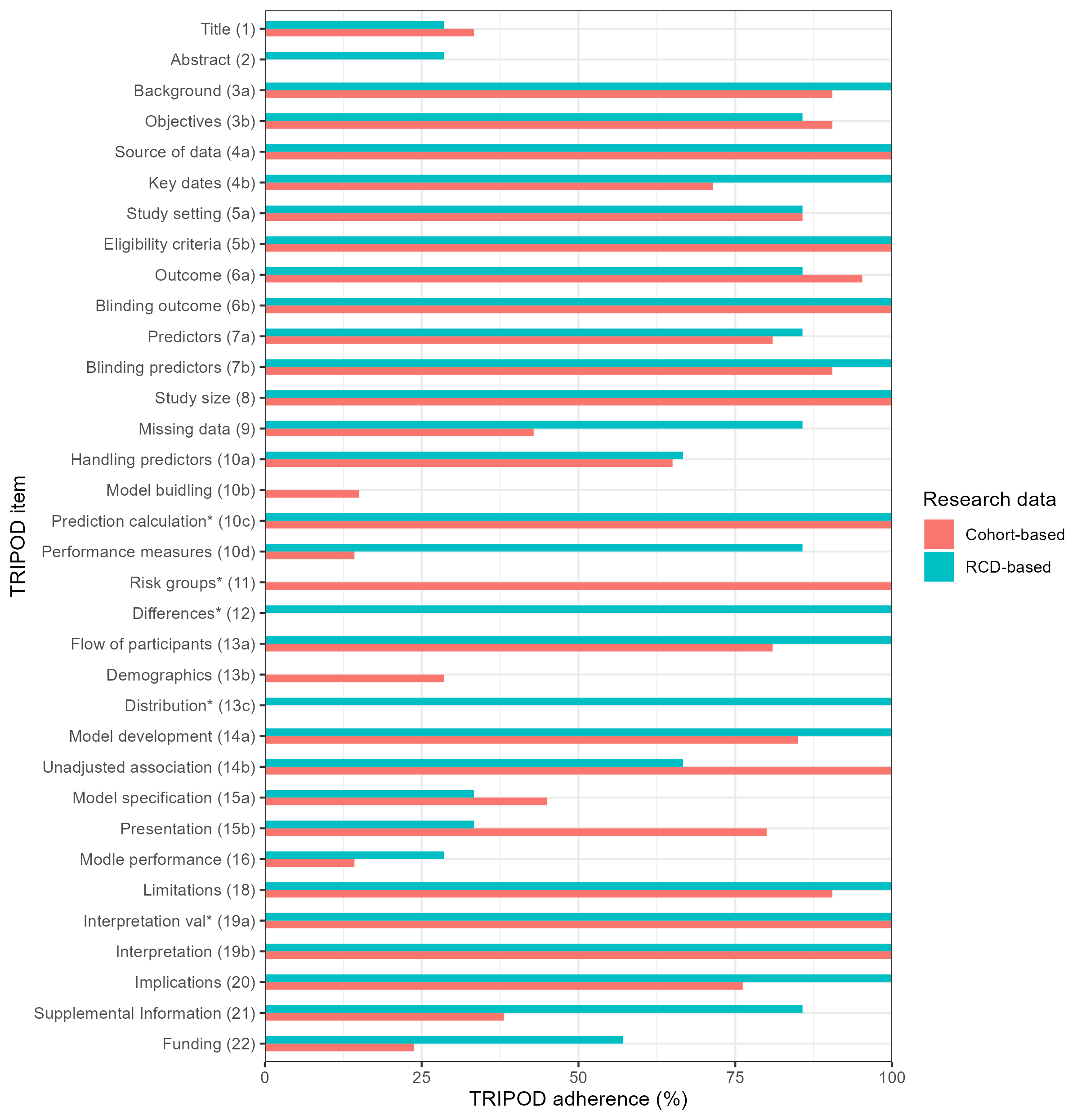


* only applies to external validation studies

# References (for appendix)

1. Dormosh N, Schut MC, Heymans MW *et al.* Development and internal validation of a risk prediction model for falls among older people using primary care electronic health records. *Journals Gerontol Ser A* 2021, DOI: 10.1093/gerona/glab311.

2. Dormosh N, Heymans MW, van der Velde N *et al.* External Validation of a Prediction Model for Falls in Older People Based on Electronic Health Records in Primary Care. *J Am Med Dir Assoc* 2022;**23**:1691-1697.e3.

3. Homer ML, Palmer NP, Fox KP *et al.* Predicting Falls in People Aged 65 Years and Older from Insurance Claims. *Am J Med* 2017;**130**:744.e17-744.e23.

4. Oshiro CES, Frankland TB, Rosales AG *et al.* Fall Ascertainment and Development of a Risk Prediction Model Using Electronic Medical Records. *J Am Geriatr Soc* 2019, DOI: 10.1111/jgs.15872.

5. Rafiq M, McGovern A, Jones S *et al.* Falls in the elderly were predicted opportunistically using a decision tree and systematically using a database-driven screening tool. *J Clin Epidemiol* 2014;**67**:877–86.

6. Smith MI, De Lusignan S, Mullett D *et al.* Predicting falls and when to intervene in older people: A multilevel logistical regression model and cost analysis. *PLoS One* 2016;**11**, DOI: 10.1371/journal.pone.0159365.

7. Ye C, Li J, Hao S *et al.* Identification of elders at higher risk for fall with statewide electronic health records and a machine learning algorithm. *Int J Med Inform* 2020;**137**, DOI: 10.1016/j.ijmedinf.2020.104105.

8. Bath PA, Pendleton N, Morgan K *et al.* New approach to risk determination: Development of risk profile for new falls among community-dwelling older people by use of a genetic algorithm neural network (GANN). *Journals Gerontol - Ser A Biol Sci Med Sci* 2000;**55**, DOI: 10.1093/gerona/55.1.M17.

9. Bongue B, Dupré C, Beauchet O *et al.* A screening tool with five risk factors was developed for fall-risk prediction in community-dwelling elderly. *J Clin Epidemiol* 2011;**64**:1152–60.

10. Cella A, de Luca A, Squeri V *et al.* Development and validation of a robotic multifactorial fall-risk predictive model: A one-year prospective study in community-dwelling older adults. Bouffanais R (ed.). *PLoS One* 2020;**15**:e0234904.

11. Coll-Planas L, Kron M, Sander S *et al.* Accidental falls among community-dwelling older adults: Improving the identification process of persons at risk by nursing staff. *Z Gerontol Geriatr* 2006;**39**:277–82.

12. Covinsky KE, Kahana E, Kercher K *et al.* History and mobility exam index to identify community-dwelling elderly persons at risk of falling. *Journals Gerontol - Ser A Biol Sci Med Sci* 2001;**56**:M253–9.

13. Delbaere K, Van den Noortgate N, Bourgois J *et al.* The physical performance test as a predictor of frequent fallers: A prospective community-based cohort study. *Clin Rehabil* 2006;**20**:83–90.

14. Deschamps T, Le Goff CG, Berrut G *et al.* A decision model to predict the risk of the first fall onset. *Exp Gerontol* 2016;**81**:51–5.

15. Ek S, Rizzuto D, Calderón-Larrañaga A *et al.* Predicting First-Time Injurious Falls in Older Men and Women Living in the Community: Development of the First Injurious Fall Screening Tool. *J Am Med Dir Assoc* 2019;**20**:1163-1168.e3.

16. Frisendahl N, Ek S, Rosendahl E *et al.* Predictive Performance of the FIF Screening Tool in 2 Cohorts of Community-Living Older Adults. *J Am Med Dir Assoc* 2020;**21**:1900-1905.e1.

17. Gade GV, Jørgensen MG, Ryg J *et al.* Development of a multivariable prognostic PREdiction model for 1-year risk of FALLing in a cohort of community-dwelling older adults aged 75 years and above (PREFALL). *BMC Geriatr* 2021;**21**:402.

18. Gadkaree SK, Sun DQ, Huang J *et al.* Comparison of Simple Versus Performance-Based Fall Prediction Models. *Gerontol Geriatr Med* 2015;**1**:233372141558485.

19. Ikeda T, Cooray U, Hariyama M *et al.* An Interpretable Machine Learning Approach to Predict Fall Risk Among Community-Dwelling Older Adults: a Three-Year Longitudinal Study. *J Gen Intern Med* 2022;**37**:2727–35.

20. Kang L, Chen X, Han P *et al.* A Screening Tool Using Five Risk Factors Was Developed for Fall-Risk Prediction in Chinese Community-Dwelling Elderly Individuals. *Rejuvenation Res* 2018;**21**:416–22.

21. Van De Loo B, Seppala LJ, Van Der Velde N *et al.* Development of the ADFICE_IT Models for Predicting Falls and Recurrent Falls in Community-Dwelling Older Adults: Pooled Analyses of European Cohorts With Special Attention to Medication. Lipsitz LA (ed.). *Journals Gerontol - Ser A Biol Sci Med Sci* 2022;**77**:1446–54.

22. Makino K, Lee S, Bae S *et al.* Simplified decision‐tree algorithm to predict falls for community‐dwelling older adults. *J Clin Med* 2021;**10**, DOI: 10.3390/jcm10215184.

23. Toba K, Kikuchi R, Iwata A. Simple screening test for risk of falls in the elderly. *Nippon rinsho Japanese J Clin Med* 2007;**65 Suppl 9**:597–601.

24. Pluijm SMF, Smit JH, Tromp EAM *et al.* A risk profile for identifying community-dwelling elderly with a high risk of recurrent falling: Results of a 3-year prospective study. *Osteoporos Int* 2006;**17**:417–25.

25. Stalenhoef PA, Diederiks JPM, Knottnerus JA *et al.* A risk model for the prediction of recurrent falls in community-dwelling elderly: A prospective cohort study. *J Clin Epidemiol* 2002;**55**:1088–94.

26. Stel VS, Pluijm SMF, Deeg DJH *et al.* A classification tree for predicting recurrent falling in community-dwelling older persons. *J Am Geriatr Soc* 2003;**51**:1356–64.

27. Tromp AM, Pluijm SMF, Smit JH *et al.* Fall-risk screening test: A prospective study on predictors for falls in community-dwelling elderly. *J Clin Epidemiol* 2001;**54**:837–44.

28. Woo J, Leung J, Wong S *et al.* Development of a simple scoring tool in the primary care setting for prediction of recurrent falls in men and women aged 65 years and over living in the community. *J Clin Nurs* 2009;**18**:1038–48.

29. Okochi J, Toba K, Takahashi T *et al.* Simple screening test for risk of falls in the elderly. *Geriatr Gerontol Int* 2006;**6**:223–7.

30. Cella A, de Luca A, Squeri V *et al.* Development and validation of a robotic multifactorial fall-risk predictive model: A one-year prospective study in community-dwelling older adults. Bouffanais R (ed.). *PLoS One* 2020;**15**:e0234904.

31. Ikeda T, Cooray U, Hariyama M *et al.* An Interpretable Machine Learning Approach to Predict Fall Risk Among Community-Dwelling Older Adults: a Three-Year Longitudinal Study. *J Gen Intern Med* 2022;**37**:2727–35.
